# High Levels of Miticides and Agrochemicals in North American Apiaries: Implications for Honey Bee Health

**DOI:** 10.1371/journal.pone.0009754

**Published:** 2010-03-19

**Authors:** Christopher A. Mullin, Maryann Frazier, James L. Frazier, Sara Ashcraft, Roger Simonds, Dennis vanEngelsdorp, Jeffery S. Pettis

**Affiliations:** 1 Department of Entomology, The Pennsylvania State University, University Park, Pennsylvania, United States of America; 2 National Science Laboratory, United States Department of Agriculture - Agricultural Marketing Service, Gastonia, North Carolina, United States of America; 3 Pennsylvania Department of Agriculture, Harrisburg, Pennsylvania, United States of America; 4 Bee Research Laboratory, United States Department of Agriculture - Agricultural Research Service, Beltsville, Maryland, United States of America; INRA - Paris 6 - AgroParisTech, France

## Abstract

**Background:**

Recent declines in honey bees for crop pollination threaten fruit, nut, vegetable and seed production in the United States. A broad survey of pesticide residues was conducted on samples from migratory and other beekeepers across 23 states, one Canadian province and several agricultural cropping systems during the 2007–08 growing seasons.

**Methodology/Principal Findings:**

We have used LC/MS-MS and GC/MS to analyze bees and hive matrices for pesticide residues utilizing a modified QuEChERS method. We have found 121 different pesticides and metabolites within 887 wax, pollen, bee and associated hive samples. Almost 60% of the 259 wax and 350 pollen samples contained at least one systemic pesticide, and over 47% had both in-hive acaricides fluvalinate and coumaphos, and chlorothalonil, a widely-used fungicide. In bee pollen were found chlorothalonil at levels up to 99 ppm and the insecticides aldicarb, carbaryl, chlorpyrifos and imidacloprid, fungicides boscalid, captan and myclobutanil, and herbicide pendimethalin at 1 ppm levels. Almost all comb and foundation wax samples (98%) were contaminated with up to 204 and 94 ppm, respectively, of fluvalinate and coumaphos, and lower amounts of amitraz degradates and chlorothalonil, with an average of 6 pesticide detections per sample and a high of 39. There were fewer pesticides found in adults and brood except for those linked with bee kills by permethrin (20 ppm) and fipronil (3.1 ppm).

**Conclusions/Significance:**

The 98 pesticides and metabolites detected in mixtures up to 214 ppm in bee pollen alone represents a remarkably high level for toxicants in the brood and adult food of this primary pollinator. This represents over half of the maximum individual pesticide incidences ever reported for apiaries. While exposure to many of these neurotoxicants elicits acute and sublethal reductions in honey bee fitness, the effects of these materials in combinations and their direct association with CCD or declining bee health remains to be determined.

## Introduction

One third of honey bee colonies in the US were lost during each of the last three winters between ’06-’09 [1]–[3]. This alarming overwinter along with other losses of this primary pollinator, *Apis mellifera* L., as well as those of native pollinators, has been documented in North America and Europe [4], [5]. The most recent manifestation of this decline, Colony Collapse Disorder (CCD), has led to a significant collaborative effort involving several land grant universities, Departments of Agriculture and the USDA. Over the past two years, the CCD working team has been investigating the possible cause(s) responsible for CCD. CCD is characterized by a rapid loss of adult bees, but not the queen and brood, along with the absence of invasive responses by robber bees and other hive pests [1].

Pesticides have long been suspected as a potential cause of honey bee declines [5], [6]. Many of these are lipophilic compounds like pyrethroids, organophosphates and associated fungicides and herbicides that can be monitored through conventional gas chromatography-mass spectrometry (GC-MS). High-value seed technologies have driven greater deployment of systemic pesticides to seasonally protect all plant organs including flowers, which inadvertently contaminates pollen and nectar. The more recently developed liquid chromatography-tandem mass spectrometry (LC/MS-MS) analytical capability is essential for monitoring systemic insecticides, like neonicotinoids [7], [8]. The enhanced sensitivity provided by LC/MS-MS allows measurement of residues at the ppb level known to affect bees sublethally, not killing them outright, but rather impairing behaviors or immune responses [9]–[11]. Other systemics such as aldicarb and its toxic metabolites, and numerous polar pesticides and their degradates could not be analyzed at ppb limits of detection without LC-MS technology [12], [13].

Since 1999, beekeepers in France experiencing bee losses described as “mad bee disease” have blamed the systemic neonicotinoid pesticide, imidacloprid [14]. Lab studies confirmed its toxicity to bees, including impaired learning and memory [10], and field studies found low levels of imidacloprid in a high percentage of pollen samples collected from maize, sunflower and canola [7], [8]. Conflicting data exist for establishing a causal relationship between imidacloprid and honey bee losses, yet regulatory concerns remain [15].

The interactions between pesticides [16], mite stresses and diseases including the newly identified Israeli acute paralysis virus [IAPV, 17] are likely contributing factors, and support an emerging hypothesis that no one factor alone is responsible for the dramatic losses of honey bees in general or for CCD specifically [18]. Only the miticide coumaphos was at consistently higher levels in non-CCD versus CCD colonies out of 50 pesticides and metabolites found [18], supporting its beneficial role in promoting apiary health by reducing *Varroa* mite stress. Pesticides have been implicated in the declines of other bioindicator species including the altering of olfactory behavior in western US salmon [19], disrupting signaling required for recruitment of nitrogen-fixing bacterial symbionts [20], and causing endocrine disruption, increased disease susceptibility, and potential declines in frogs and other amphibian species through synergistic interactions with chytrid fungi [21], [22]. A potential involvement of pesticides remains to be investigated in eliciting the “white-nose syndrome” that is decimating northeastern US bat populations [23].

During 2007 to 2008, we actively sampled beebread, trapped pollen, brood nest wax, beeswax foundation, and adult bees and brood for pesticide residues. These samples were drawn largely from commercial beekeepers from several states and one Canadian province, and included samples from apparently healthy colonies as well as from operations that were diagnosed as having CCD. Included in this survey were dead bees collected from local or community applications of insecticides. A comprehensive and sensitive analytical survey of 200 miticides, insecticides, fungicides and herbicides was conducted, including some no longer registered for use, to broadly assess known bee toxicants and other likely co-occurring pesticides. Here we document the plethora of pesticides that are currently present in US beehives and discuss their potential risks to honey bee health.

## Materials and Methods

### Beehive samples

In 2007 and 2008 we analyzed pollen (total of 320 beebread, 28 trapped pollen, and 2 anther samples), 238 wax (derived mainly from the brood nest of colonies) and 21 foundation samples, and 34 immature (brood) and 106 adult bee samples for pesticide residues. These samples were collected as part of different studies and epidemiological surveys to investigate possible threats to colony health. The studies and surveys are described here. In January and February 2007, colonies resident in Florida and California distributed across 13 apiaries owned by 11 different beekeepers were selected to participate in multi-factorial study. Apiaries were classified as 1) having no colonies with CCD symptoms (‘control’) or 2) having colonies with CCD symptoms (‘CCD ’). Colonies were considered to have CCD symptoms when adult bee populations were in obvious decline leaving brood poorly attended, or were dead in an apiary having clear symptoms of CCD. In those CCD colonies where bees remained, there were insufficient number of bees to cover the brood, the remaining worker bees appeared young (i.e., adults bees that are unable to fly), and the queen was present. In a second study to investigate pesticides exposure to honey bee colonies engaged in apple pollination, samples of pollen, wax and bees were collected from 47 colonies in 2007 and 2008. These colonies were distributed in three Pennsylvania apple orchards with known pesticide application histories and a control location. In 2007, a longitudinal study was conducted which followed tagged colonies in three migratory operations as they moved from Florida up and down the east coast to pollinate a variety of crops (citrus, high bush blueberries, low bush blueberries, apples, cucumbers, squash, pumpkin). Samples of pollen, wax, and adult bees, and detailed colony measurements were taken each time these colonies were moved to a new crop. In this last survey [24] a new phenomenon, entombed and capped pollen, was observed, and samples of these pollens, plus respective wax, were included here. In these three studies, samples were collected by researchers from the CCD working group. In 2008, 65 of the pollen, wax, adult bee and honey samples were submitted for analysis directly by beekeepers from 13 different states as part of a program to share the cost of analysis.

In some cases sampled colonies had a ∼15 cm by 10 cm section of brood comb removed and wrapped in aluminum foil and stored on dry ice until placed in a −80°C freezer. These sections of comb contained beeswax, beebread and brood. Beebread and brood were removed from the combs at room temperature and then stored along with the remaining beeswax at −20°C until processing. In other cases, samples of beebread collected in the field were removed from the brood nest of colonies using a spatula cleaned using Clorox® wipes and rinsed with 75% ethanol between collections. Beebread was placed in a 1.5 ml Eppendorf tube on dry ice until storage at −20°C. Samples of brood nest wax collected in the field were scraped with a sterilized standard hive tool into a 50 ml centrifuge tube and similarly stored. While care was taken to sample sections of the comb without honey, nectar, beebread or brood, small levels of cross-contamination were inevitable. Adult nurse bees were removed from the brood nest and placed into 50 ml centrifuge tubes on dry ice until they could be stored at −80°C.

In the apple orchard study, samples were field collected as described above but were placed on ice after collection and then stored in a standard freezer (−20°C). Beekeepers submitting samples were provided with a standardized protocol for collecting, storing and shipping samples. They were instructed to freeze all samples as soon as possible after collection and then ship samples overnight or second-day delivery in insulated containers with ice packs. Upon arrival these samples were stored in a standard freezer.

Foundation is processed beeswax pressed into sheets and used as templates for uniform comb construction. Wax samples from six different commercial and two private sources were analyzed. This included one sample of wax from wax-coated plastic foundation.

The majority of samples (749) we analyzed included brood nest wax and foundation, pollen and bees from colonies associated with the specific research projects described above. While the sampling was not completely random across time and space, it does include migratory or stationary colonies diagnosed as having “CCD” as well as those diagnosed as healthy, colonies placed in orchards with known pesticide application history as well as control colonies not placed in orchards, and samples submitted by beekeeper from colonies described as “unhealthy” as well as from those identified as “healthy.” The results and conclusion reported here are drawn mainly from these data. In addition, we analyzed 158 samples that included mixed matrices (pollen and wax), *Osmia-*collected pollen, floral nectar, hive supplements (corn syrup, pollen substitute), royal jelly, honey, samples obtained outside of U.S. and Canada and irradiated samples. Residue data on these samples are included in **Table S1**.

### Multiresidue pesticide analysis

Samples over the entire study were analyzed for 200 chemicals at an average of 171 pesticides and toxic metabolites per analysis. New compounds were added and others removed depending on lack of detection or negligible frequency of use where bees forage. Pesticide residue analysis was conducted by the USDA-AMS-NSL at Gastonia, NC. For multi-residue pesticide analysis, a modified QuEChERS method was used [25] that was adapted for 3 g instead of the normal 15 g samples. Beebread or comb wax (3 g) is weighed into a 50 ml plastic centrifuge tube and fortified with 100 µl of the process control spiking (PCS) solution. After adding 27 ml of extraction solution (44% deionized water, 55% acetonitrile, and 1% glacial acetic acid), each sample is then fortified with 100 µl of the internal standard (ISTD) spiking solution. For beebread, the particle size is reduced by using a high speed disperser for approximately 1 minute. For comb wax, the sample is melted and dispersed by heating to 80°C for 20 min in a water bath, followed by cooling to room temperature. To each sample is then added 6 g of anhydrous magnesium sulfate (MgSO_4_) and 1.5 g anhydrous sodium acetate (NaAc). Tubes are sealed and shaken vigorously for 1minute, centrifuged, and 1 ml of supernatant **A** or its concentrate transferred to a 2 ml mini-centrifuge tube that contains 0.05 g primary secondary amine (PSA), 0.05 g C18, and 0.15 g MgSO_4_ (United Chemical Technologies, Lewistown, PA). After vortexing for 1 minute and centrifugation, the resulting supernatant is transferred to an autosampler vial for analysis by LC/MS-MS using a 3.5 µm, 2.1×150 mm Agilent Zorbax SB-C18 column and an Agilent 1100 LC with a binary pump interfaced to a Thermo-Fisher TSQ Quantum Discovery triple quadrupole MS.

For GC analyses, a dual layer solid-phase extraction (SPE) cartridge containing 250 mg of graphitized carbon black (GCB) and 500 mg of PSA is prepared with approximately 0.80 g of anhydrous MgSO_4_ added to the top of the cartridge. After conditioning the SPE cartridge by adding one cartridge volume (4.0 ml) of acetone/toluene (7∶3; v/v) using a positive pressure SPE manifold and eluting to waste, 2 ml of supernatant **A** (above) is applied to the cartridge. Pesticide analytes are eluted with 3 by 4 ml of acetone/toluene (7∶3; v/v) into a 15 ml graduated glass centrifuge tube. Using an N-Evap at 50°C, eluates are dried using toluene and concentrated to a final volume of 0.4 ml for analysis using GC/MS in the electron impact and negative chemical ionization modes. An Agilent 6890 GC equipped with a 0.25 mm id×30 m J&W DB-5MS (2 µm film) capillary column interfaced to an Agilent 5975 triple quadrupole MS was used. A parallel method was used for the brood and adult bee matrices, except that water was deleted from the extraction solution due to its high content in the samples.

Extracts of wax, beebread, and adult bees or brood were also analyzed for potentially toxic metabolites of primary miticide and insecticide detections. This included the respective oxon and the phenolic metabolite of coumaphos, chlorferone, the sulfoxide and sulfone metabolites of aldicarb, and the toxic olefin and 5-hydroxy metabolites of imidacloprid. Pesticides and metabolites were obtained in high-purity as standards from the EPA, Chem Service (West Chester, PA), or the manufacturer at the highest purity available.

Identity of parent pesticides and metabolites from extracts was based on co-chromatography with known standards by GC/MS and/or LC/MS-MS and consistent ratios of parent mass abundance to at least two fragment transitions. Standard parent mass and fragment ion transitions used [12] are also available online [26]. A matrix-dependent limit of detection (LOD) for each parent and metabolite was determined after adjustment for recovery of the ISTD.

### Bee toxicity

Honey bee LD_50_ values are averaged 24–72 h adult acute toxicities from the EPA-OPP Pesticide Ecotoxicity Database [http://www.ipmcenters.org/Ecotox/DataAccess.cfm] and primary literature [27]–[29]. Standard LD_50_ values in terms of µg/bee were converted to ppb relative to body weight (ng pesticide per g bee) by multiplying using a factor of 10,000; equivalent to1000 ng per µg ÷ average bee weight of 0.1 g.

### Statistical analyses

Mean, medians, percentiles, and standard errors of the means for individual pesticides and metabolites for all matrix-specific or paired pesticide analyses were calculated using 0 ppb for any non-detection (N.D.), unless otherwise noted. In-hive and between colony comparisons of pesticide detections were made by pairing 749 bee, pollen and wax sample analyses by colony/matrix, and then sorting colonies for concurrently-sampled matrices. This paired database of 519 analyses was further averaged according to matrix by colony identity if sampling dates were not identical. Significant trends were extracted by correlation followed by linear regression analysis of these data using Microsoft Excel Data Analysis package (ver. 11.5) or SAS JMP ver. 9.0. A two sample one way ANOVA was used to determine significant differences between compounds or treatments at the P<0.05 level.

## Results

### Honey bees across North America are extensively exposed to multiple pesticides

Brood nest wax and foundations, beebread and trapped pollen, and adult bees and brood comprising 749 samples contained 118 different pesticides and metabolites, 4894 total residues of which 748 were systemics, and averaged 6.5 detections per sample. In the 259 wax samples (**Table 1**) 87 pesticides and metabolites were found with up to 39 different detections in a single sample, averaging 8 different pesticide residues each. In the 350 pollen samples analyzed (**Table 2**), 98 pesticides and degradates were identified, with up to 31 different pesticides found in a single sample, and samples averaged 7.1 different pesticide residues each. The analysis of bees resulted in fewer detections (**Table 3**), and averaged 2.5 residues per each of the 140 samples, with a maximum of 25 in one sample. Only one of the wax, three pollen and 12 bee samples had no detectable pesticides.

**Table 1:** Summary of pesticide detections in wax samples from North American honey bee colonies.

Wax Pesticide*	Class#	Detects	Samples	%	Detections (ppb)							
			Analyzed		High	Low	Median	90%tile	95%tile	Mean§	SEM§	LOD†
Fluvalinate	PYR	254	259	98.1	204000.0	2.0	3595.0	15080.0	28710.5	7473.8	973.6	1.0
Coumaphos	OP	254	259	98.1	91900.0	1.0	1240.0	6875.0	11340.0	3300.4	499.8	1.0
Coumaphos oxon	OP	187	208	89.9	1300.0	1.3	56.1	184.2	269.8	102.7	12.5	5.0
Chlorpyrifos	OP	163	258	63.2	890.0	1.0	4.3	28.5	55.7	24.5	7.5	0.1
Chlorothalonil	FUNG	127	258	49.2	53700.0	1.0	91.4	1552.0	2623.0	1066.6	453.4	1.0
DMPF (amitraz)	FORM	107	177	60.5	43000.0	9.2	228.0	4718.0	8093.0	2199.8	574.2	4.0
Endosulfan I	CYC	97	258	37.6	95.0	1.2	4.1	13.0	31.0	8.7	1.5	0.1
Endosulfan II	CYC	65	258	25.2	39.0	1.1	3.8	10.9	21.2	6.2	0.8	0.1
DMA (amitraz)	FORM	60	177	33.9	3820.0	120.0	437.0	1664.0	2433.0	742.1	104.6	50.0
Pendimethalin	HERB	49	176	27.8	84.0	2.5	6.1	18.7	36.0	10.9	2.1	1.0
Fenpropathrin	PYR	44	258	17.1	200.0	1.3	14.3	51.3	61.3	24.8	5.0	0.4
Esfenvalerate	PYR	43	258	16.7	56.1	1.0	4.5	17.0	19.9	8.9	1.5	0.5
Azoxystrobin	S FUNG	40	258	15.5	278.0	1.0	5.7	22.4	40.4	15.4	6.9	1.0
Methoxyfenozide	IGR	39	208	18.8	495.0	3.5	42.3	171.0	271.4	81.5	17.2	0.4
Bifenthrin	PYR	33	258	12.8	56.1	1.5	5.3	18.5	39.5	9.8	2.3	0.4
Endosulfan sulfate	CYC	29	258	11.2	33.0	1.3	3.0	12.1	18.4	6.3	1.3	0.1
Atrazine	S HERB	29	208	13.9	31.0	1.0	5.5	16.5	18.4	8.2	1.3	1.0
Dicofol	OC	26	258	10.1	21.0	1.5	5.1	15.1	17.5	6.8	1.1	0.4
Aldicarb sulfoxide	S CARB	22	208	10.6	649.0	13.4	298.5	609.2	638.8	306.6	48.0	20.0
Trifluralin	HERB	22	176	12.5	36.0	1.0	1.4	2.2	21.0	3.9	1.8	1.0
Boscalid	S FUNG	21	208	10.1	388.0	16.9	84.0	261.0	265.0	109.8	20.6	1.0
Carbendazim	S FUNG	21	208	10.1	133.0	2.1	12.0	48.7	87.0	23.2	7.0	1.0
Oxyfluorfen	HERB	16	258	6.2	34.0	2.1	6.1	26.5	29.1	11.1	2.6	0.5
Methidathion	OP	15	258	5.8	78.7	2.9	10.0	23.0	40.5	15.3	4.8	1.0
Aldicarb sulfone	S CARB	15	208	7.2	49.6	18.0	27.5	45.8	48.1	31.0	2.8	10.0
Iprodione	FUNG	14	208	6.7	636.0	32.6	164.5	555.2	586.6	269.7	52.4	10.0
Pyrethrins	PYR	13	208	6.3	222.0	19.0	78.7	151.4	181.2	84.5	16.9	20.0
Cypermethrin	PYR	13	258	5.0	131.0	4.5	13.2	95.3	114.8	31.2	11.4	1.0
Norflurazon	S HERB	13	208	6.3	38.1	1.1	2.9	5.6	18.7	5.8	2.7	1.0
Vinclozolin	FUNG	13	258	5.0	27.0	1.2	4.6	21.7	24.6	8.8	2.4	1.0
Cyhalothrin	PYR	13	258	5.0	16.9	1.0	5.7	13.2	15.3	6.5	1.3	0.1
Chlorferone (coumaphos)	OP	11	176	6.3	4390.0	299.0	932.0	2830.0	3610.0	1236.7	381.6	25.0
Cyprodinil	S FUNG	11	208	5.3	106.0	6.2	17.0	85.4	95.7	34.7	10.3	5.0
Cyfluthrin	PYR	11	258	4.3	44.7	3.2	7.8	17.0	30.9	12.6	3.5	1.0
Pyraclostrobin	FUNG	10	208	4.8	438.0	1.8	27.3	193.2	315.6	84.2	42.4	1.0
Fenbuconazole	S FUNG	10	176	5.7	183.0	7.4	46.1	86.0	134.5	54.2	15.7	6.0
Tebufenozide	IGR	10	208	4.8	27.7	2.0	5.3	18.3	23.0	8.0	2.6	2.0
Pronamide	S HERB	10	208	4.8	22.8	1.7	3.0	12.5	17.6	6.1	2.1	1.0
Deltamethrin	PYR	8	258	3.1	613.0	107.0	129.5	368.0	490.5	209.9	60.6	20.0
Allethrin	PYR	8	208	3.8	139.0	1.7	9.2	62.1	100.5	28.0	16.1	1.0
Trifloxystrobin	PS FUNG	8	258	3.1	22.4	2.6	4.2	12.0	17.2	6.7	2.3	0.5
Azinphos methyl	OP	6	258	2.3	121.0	10.9	18.8	75.0	98.0	35.2	17.4	3.0
Tribufos = DEF	SYN	6	208	2.9	59.0	7.6	19.3	44.1	51.5	25.1	7.4	2.0
Malathion	OP	6	258	2.3	35.1	4.0	5.2	26.6	30.8	12.1	5.1	1.0
*p*-Dichlorobenzene	OC	5	130	3.8	1050.0	6.9	30.9	642.7	846.3	228.0	205.5	6.0
Permethrin	PYR	5	258	1.9	372.0	31.0	227.8	333.6	352.8	209.6	58.2	10.0
Phosmet	OP	5	258	1.9	209.0	2.9	28.3	157.8	183.4	69.0	37.3	2.0
DDE p,p'	OC	5	208	2.4	31.0	5.5	11.3	30.9	31.0	17.2	5.7	3.0
Flutolanil	S FUNG	4	208	1.9	105.0	7.2	54.2	102.1	103.5	55.2	26.1	4.0
Thiacloprid	S NEO	4	208	1.9	7.8	1.9	5.9	7.5	7.7	5.4	1.3	1.0
Diazinon	OP	4	208	1.9	4.3	1.4	1.6	3.5	3.9	2.2	0.7	1.0
Thiabendazole	S FUNG	3	208	1.4	76.0	7.4	19.0	64.6	70.3	34.1	21.2	1.0
Fipronil	INS	3	208	1.4	35.9	1.1	1.3	29.0	32.4	12.8	11.6	1.0
Dieldrin	CYC	3	258	1.2	35.4	6.9	12.1	30.7	33.1	18.1	8.8	4.0
Pyrimethanil	FUNG	3	208	1.4	27.8	3.4	11.7	24.6	26.2	14.3	7.2	2.0
Tebuthiuron	S HERB	3	208	1.4	22.4	4.9	5.8	19.1	20.7	11.0	5.7	1.0
Chlorfenapyr	PS MITI	3	176	1.7	11.9	1.3	3.6	10.2	11.1	5.6	3.2	1.0
Parathion methyl	OP	3	208	1.4	6.1	3.8	4.0	5.7	5.9	4.6	0.7	1.0
Quintozene = PCNB	FUNG	3	208	1.4	2.5	1.0	1.3	2.3	2.4	1.6	0.5	1.0
Ethofumesate	S HERB	2	208	1.0	560.0	224.0	392.0	526.4	543.2	392.0	168.0	5.0
Propiconazole	S FUNG	2	208	1.0	227.0	166.0	196.5	220.9	224.0	196.5	30.5	3.0
Piperonyl butoxide	SYN	2	208	1.0	208.0	31.1	119.6	190.3	199.2	119.6	88.5	6.0
Dimethomorph	S FUNG	2	176	1.1	133.0	58.0	95.5	125.5	129.3	95.5	37.5	15.0
Ethion	OP	2	208	1.0	131.0	83.6	107.3	126.3	128.6	107.3	23.7	2.0
Captan	FUNG	2	258	0.8	69.1	25.0	47.1	64.7	66.9	47.1	22.1	10.0
Fluoxastrobin	S FUNG	2	208	1.0	44.5	23.1	33.8	42.4	43.4	33.8	10.7	4.0
Bendiocarb	S CARB	2	257	0.8	22.0	5.5	13.8	20.4	21.2	13.8	8.3	2.0
Carbofuran, 3-hydroxy	S CARB	2	208	1.0	21.1	12.4	16.8	20.2	20.7	16.8	4.4	3.0
Carfentrazone ethyl	PS HERB	2	208	1.0	17.0	4.9	11.0	15.8	16.4	11.0	6.1	1.0
Imidacloprid	S NEO	2	208	1.0	13.6	2.4	8.0	12.5	13.0	8.0	5.6	2.0
Tetradifon	MITI	2	208	1.0	11.1	4.7	7.9	10.5	10.8	7.9	3.2	1.0
Metribuzin	S HERB	2	208	1.0	8.0	1.0	4.5	7.3	7.7	4.5	3.5	1.0
Pyriproxyfen	IGR	2	208	1.0	7.6	2.2	4.9	7.1	7.3	4.9	2.7	1.0
Prallethrin	PYR	2	208	1.0	6.8	4.3	5.6	6.6	6.7	5.6	1.3	4.0
Fluridone	S HERB	2	208	1.0	6.6	5.7	6.2	6.5	6.6	6.2	0.4	5.0
Fenamidone	FUNG	1	208	0.5	138.0	138.0	138.0	138.0	138.0	138.0	---	10.0
Heptachlor	CYC	1	208	0.5	31.0	31.0	31.0	31.0	31.0	31.0	---	4.0
Spirodiclofen	MITI	1	208	0.5	28.5	28.5	28.5	28.5	28.5	28.5	---	1.0
Heptachlor epoxide	CYC	1	208	0.5	13.3	13.3	13.3	13.3	13.3	13.3	---	1.0
Fenhexamid	FUNG	1	176	0.6	9.3	9.3	9.3	9.3	9.3	9.3	---	5.0
Carbofuran	S CARB	1	208	0.5	5.8	5.8	5.8	5.8	5.8	5.8	---	5.0
Pyridaben	MITI	1	208	0.5	5.4	5.4	5.4	5.4	5.4	5.4	---	1.0
Carbaryl	PS CARB	1	208	0.5	4.5	4.5	4.5	4.5	4.5	4.5	---	5.0
Tefluthrin	PYR	1	208	0.5	3.3	3.3	3.3	3.3	3.3	3.3	---	1.0
Triadimefon	S FUNG	1	208	0.5	2.4	2.4	2.4	2.4	2.4	2.4	---	2.0
Metalaxyl	S FUNG	1	208	0.5	1.4	1.4	1.4	1.4	1.4	1.4	---	1.0
Hexachlorobenzene	FUNG	1	258	0.4	1.0	1.0	1.0	1.0	1.0	1.0	---	0.1

*Carbendazim is also a degradate of benomyl; Thiabendazole is a degradate of thiophanate methyl.

# Class: CAR  =  carbamate, CYC  =  cyclodiene, FORM  =  formamidine, FUNG  =  fungicide, HERB  =  herbicide, IGR  =  insect growth regulator, INS  =  misc. insecticide, MITI  =  miticide, NEO  =  neonicotinoid, OC  =  organochlorine, OP  =  organophosphate, PS  =  partial systemic, PYR  =  pyrethroid, S  =  systemic.

§ Mean and SEM for detections > LOD.

† LOD  =  limit of detection (ppb).

**Table 2:** Summary of pesticide detections in pollen samples from North American honey bee colonies.

Pollen Pesticide*	Class#	Detects	Samples	%	Detections (ppb)							
					High	Low	Median	90%tile	95%tile	Mean§	SEM§	LOD^†^
Fluvalinate	PYR	309	350	88.3	2670.0	1.6	40.2	186.8	323.0	95.1	12.6	1.0
Coumaphos	OP	263	350	75.1	5828.0	1.0	13.1	518.4	892.0	180.4	33.0	1.0
Chlorpyrifos	OP	153	350	43.7	830.0	0.1	4.4	140.4	226.5	53.3	10.6	0.1
Chlorothalonil	FUNG	148	280	52.9	98900.0	1.1	35.0	9939.0	18765.0	3014.8	880.9	1.0
Pendimethalin	HERB	113	247	45.7	1730.0	1.1	13.4	72.9	129.8	44.6	15.7	1.0
Endosulfan I	CYC	98	350	28.0	76.7	0.4	4.2	33.9	47.2	10.9	1.5	0.1
Endosulfan sulfate	CYC	92	350	26.3	35.0	0.2	2.2	9.2	11.3	4.3	0.6	0.1
DMPF (amitraz)	FORM	77	247	31.2	1117.0	6.1	75.0	360.2	615.0	147.9	23.5	4.0
Atrazine	S HERB	71	350	20.3	49.0	4.2	8.9	27.0	35.2	13.6	1.1	1.0
Endosulfan II	CYC	70	350	20.0	67.7	0.1	3.8	24.7	39.6	9.1	1.6	0.1
Fenpropathrin	PYR	63	350	18.0	170.0	0.4	7.0	24.6	60.8	15.1	3.3	0.4
Azoxystrobin	S FUNG	53	350	15.1	107.0	1.0	10.2	58.9	68.1	21.0	3.3	1.0
Metolachlor	PS HERB	52	350	14.9	103.0	2.6	8.1	19.4	44.6	13.4	2.5	2.0
Captan	FUNG	45	350	12.9	10000.0	16.0	103.0	571.8	663.2	433.5	219.9	10.0
Esfenvalerate	PYR	41	350	11.7	59.6	1.0	3.3	10.0	47.5	7.8	2.2	0.5
Carbaryl	PS CARB	38	350	10.9	1010.0	13.6	36.7	269.5	602.9	117.1	36.5	5.0
Cyhalothrin	PYR	38	350	10.9	28.0	0.1	1.7	4.3	18.2	3.4	0.9	0.1
THPI (captan)	PS FUNG	35	247	14.2	363.0	60.1	227.0	312.0	342.0	205.8	15.1	30.0
Methoxyfenozide	IGR	29	350	8.3	128.0	0.4	22.3	96.4	111.2	35.0	7.1	0.4
Dicofol	OC	28	350	8.0	143.0	0.4	8.1	60.3	85.7	23.2	6.4	0.4
Trifloxystrobin	PS FUNG	27	350	7.7	264.0	0.6	10.3	96.2	168.4	34.1	11.9	0.5
Tebufenozide	IGR	27	350	7.7	58.4	2.0	12.5	28.9	30.0	14.8	2.4	2.0
Diazinon	OP	27	350	7.7	29.0	1.0	4.6	25.6	27.7	9.2	1.8	1.0
Cypermethrin	PYR	25	350	7.1	49.0	1.6	4.6	27.8	44.7	10.8	2.6	1.0
Cyfluthrin	PYR	24	350	6.9	33.6	1.1	5.1	9.9	9.9	6.7	1.3	1.0
Azinphos methyl	OP	23	350	6.6	643.0	3.9	22.0	104.7	615.3	86.2	37.1	3.0
Aldicarb sulfoxide	S CARB	21	350	6.0	1245.0	22.0	327.0	1039.0	1146.0	493.7	85.7	20.0
Phosmet	OP	20	350	5.7	418.0	3.7	38.0	284.2	351.7	110.0	28.3	2.0
Thiacloprid	S NEO	19	350	5.4	115.0	1.7	14.0	42.9	108.7	23.8	7.2	1.0
Pyrimethanil	FUNG	19	350	5.4	83.0	2.0	8.3	67.6	82.2	18.5	6.0	2.0
Norflurazon	S HERB	18	350	5.1	108.0	2.8	23.5	54.3	66.9	29.7	6.0	1.0
1-Naphthol (carbaryl)	S CARB	18	350	5.1	85.5	3.6	9.7	45.6	58.7	20.9	5.2	2.0
Metribuzin	S HERB	18	350	5.1	44.0	1.0	3.3	10.1	15.6	6.3	2.3	1.0
Bifenthrin	PYR	18	350	5.1	12.6	0.7	3.0	7.1	7.6	3.9	0.7	0.4
Carbendazim	S FUNG	16	350	4.6	149.0	1.5	4.5	46.0	89.0	18.8	9.7	1.0
Cyprodinil	S FUNG	15	350	4.3	344.0	5.3	18.7	246.8	286.6	90.2	29.1	5.0
Myclobutanil	S FUNG	14	350	4.0	981.0	4.4	72.8	565.6	798.4	192.3	78.3	2.0
Propiconazole	S FUNG	14	350	4.0	361.0	3.1	68.0	203.2	259.6	110.3	27.3	3.0
Fenbuconazole	S FUNG	14	247	5.7	264.0	11.0	55.4	174.9	217.8	80.6	19.9	6.0
Coumaphos oxon	OP	14	280	5.0	89.0	5.4	13.5	38.2	52.2	21.2	5.9	5.0
Methidathion	OP	14	350	4.0	32.7	7.8	21.0	31.7	32.3	21.6	2.1	1.0
Malathion	OP	13	350	3.7	61.0	0.9	5.9	16.2	35.2	10.4	4.4	1.0
Aldicarb sulfone	S CARB	12	350	3.4	97.2	17.0	43.8	87.7	93.2	46.8	7.8	10.0
Simazine	S HERB	12	350	3.4	54.0	5.2	22.0	36.9	44.7	22.4	4.3	5.0
Pronamide	S HERB	11	350	3.1	378.0	17.7	71.0	355.0	366.5	122.9	38.7	1.0
Indoxacarb	INS	11	350	3.1	330.0	10.0	102.0	175.0	252.5	118.2	24.7	10.0
Acetamiprid	S NEO	11	350	3.1	134.0	14.0	57.0	101.0	117.5	59.3	11.8	5.0
Deltamethrin	PYR	11	350	3.1	91.0	28.0	66.0	88.5	88.4	66.6	6.2	20.0
Imidacloprid	S NEO	10	350	2.9	206.0	6.2	20.5	63.0	41.3	39.0	19.0	2.0
Fenhexamid	FUNG	9	247	3.6	129.0	5.8	28.0	53.8	96.1	34.4	12.3	5.0
Permethrin	PYR	9	350	2.6	92.0	9.6	28.7	89.6	73.8	40.1	10.7	10.0
Trifluralin	HERB	9	247	3.6	14.4	1.0	1.9	10.9	12.6	3.9	1.6	1.0
Tebuthiuron	S HERB	8	350	2.3	48.0	1.6	16.2	34.7	40.4	17.9	5.8	1.0
Thiabendazole	S FUNG	8	350	2.3	5.6	1.4	2.4	4.8	5.2	3.0	0.5	1.0
Dimethomorph	S FUNG	7	247	2.8	166.0	17.2	25.2	95.1	142.4	46.9	20.2	15.0
Oxyfluorfen	HERB	7	350	2.0	4.5	0.5	1.8	3.1	3.8	2.0	0.5	0.5
Difenoconazole	S FUNG	6	350	1.7	214.1	48.3	122.4	184.8	199.4	129.8	22.2	10.0
Famoxadone	FUNG	6	350	1.7	141.0	73.5	95.7	125.5	133.3	98.3	10.9	20.0
Diphenylamine	FUNG	6	103	5.8	32.0	3.6	10.5	24.5	28.3	13.2	4.3	2.0
Hexachlorobenzene	FUNG	6	350	1.7	0.4	0.1	0.2	0.4	0.4	0.2	0.1	0.1
Pyridaben	MITI	5	350	1.4	26.6	10.9	19.0	25.6	26.1	18.8	3.0	1.0
Diflubenzuron	IGR	4	350	1.1	128.0	15.0	78.5	122.0	125.0	75.0	26.1	10.0
Oxamyl	S CARB	4	350	1.1	43.0	20.0	32.5	40.3	41.7	32.0	4.7	5.0
Allethrin	PYR	4	350	1.1	11.0	6.6	7.9	10.2	10.6	8.3	0.9	1.0
Vinclozolin	FUNG	4	350	1.1	4.1	1.0	1.0	3.2	3.6	1.8	0.8	1.0
Boscalid	S FUNG	3	350	0.9	962.0	1.4	12.0	772.0	11.5	325.1	318.4	1.0
Potasan (coumaphos)	OP	3	247	1.2	160.0	61.5	138.0	155.6	157.8	119.8	29.9	10.0
Pyrethrins	PYR	3	350	0.9	61.5	27.0	35.0	56.2	61.5	41.2	10.4	20.0
Tebuconazole	S FUNG	3	350	0.9	34.0	6.4	18.0	30.8	32.4	19.5	8.0	3.0
Prallethrin	PYR	3	350	0.9	7.6	4.7	7.3	7.5	7.6	6.5	0.9	4.0
Carfentrazone ethyl	PS HERB	3	350	0.9	2.5	1.5	2.4	2.5	2.5	2.1	0.3	1.0
Propanil	HERB	2	350	0.6	358.0	265.0	311.5	348.7	353.4	311.5	46.5	10.0
Pyraclostrobin	FUNG	2	350	0.6	265.0	26.6	145.8	241.2	26.6	145.8	119.2	1.0
DDT p,p'	OC	2	350	0.6	35.6	6.0	20.8	32.6	34.1	20.8	14.8	2.0
Fluridone	S HERB	2	350	0.6	24.0	5.8	14.9	22.2	22.2	14.9	9.1	5.0
DDD p,p'	OC	2	350	0.6	13.4	11.8	12.6	13.2	13.3	12.6	0.8	4.0
4,4-dibromobenzophenone	MITI	2	247	0.8	10.8	2.2	6.5	9.9	10.4	6.5	4.3	2.0
Carbofuran, 3-hydroxy	S CARB	2	350	0.6	4.6	3.6	4.1	4.5	4.6	4.1	0.5	3.0
DDE p,p'	OC	2	350	0.6	4.3	3.3	3.8	4.2	4.3	3.8	0.5	3.0
Chlorfenapyr	PS MITI	2	247	0.8	1.4	1.2	1.3	1.4	1.4	1.3	0.1	1.0
Diphenamid	S FUNG	2	350	0.6	1.0	1.0	1.0	1.0	1.0	1.0	0.0	1.0
Imidacloprid olefin	S NEO	1	350	0.3	554.0	554.0	554.0	554.0	554.0	554.0	---	25.0
Sethoxydim	S HERB	1	350	0.3	173.0	173.0	173.0	173.0	173.0	173.0	---	1.0
Acephate	S OP	1	350	0.3	163.0	163.0	163.0	163.0	163.0	163.0	---	35.0
Imidacloprid, 5-hydroxy	S NEO	1	350	0.3	152.0	152.0	152.0	152.0	152.0	152.0	---	25.0
Amicarbazone	HERB	1	350	0.3	98.0	98.0	98.0	98.0	98.0	98.0	---	30.0
Phenothrin	PYR	1	350	0.3	83.9	83.9	83.9	83.9	83.9	83.9	---	10.0
Fenamidone	FUNG	1	350	0.3	73.9	73.9	73.9	73.9	73.9	73.9	---	10.0
Thiamethoxam	S NEO	1	350	0.3	53.3	53.3	53.3	53.3	53.3	53.3	---	5.0
Phosalone	OP	1	247	0.4	31.3	31.3	31.3	31.3	31.3	31.3	---	10.0
Fipronil	INS	1	350	0.3	28.5	28.5	28.5	28.5	28.5	28.5	---	1.0
Chlorfenvinphos	OP	1	247	0.4	10.7	10.7	10.7	10.7	10.7	10.7	---	6.0
Iprodione	FUNG	1	350	0.3	10.3	10.3	10.3	10.3	10.3	10.3	---	10.0
Spiromesifen	S INS	1	350	0.3	10.0	10.0	10.0	10.0	10.0	10.0	---	10.0
Tetramethrin	PYR	1	350	0.3	6.1	6.1	6.1	6.1	6.1	6.1	---	6.0
Tribufos = DEF	SYN	1	350	0.3	3.5	3.5	3.5	3.5	3.5	3.5	---	2.0
Spirodiclofen	MITI	1	350	0.3	1.9	1.9	1.9	1.9	1.9	1.9	---	1.0
Heptachlor epoxide	CYC	1	350	0.3	1.7	1.7	1.7	1.7	1.7	1.7	---	1.0

*Carbendazim is also a degradate of benomyl; Thiabendazole is a degradate of thiophanate methyl.

# Class: CAR  =  carbamate, CYC  =  cyclodiene, FORM  =  formamidine, FUNG  =  fungicide, HERB  =  herbicide, IGR  =  insect growth regulator, INS  =  misc. insecticide, MITI  =  miticide, NEO  =  neonicotinoid, OC  =  organochlorine, OP  =  organophosphate, PS  =  partial systemic, PYR  =  pyrethroid, S  =  systemic.

§ Mean and SEM for detections > LOD. **^†^**LOD  =  limit of detection (ppb).

**Table 3:** Summary of pesticide detections in bees from North American honey bee colonies.

Bee Pesticide*	Class#	Detects	Samples	%	Detections (ppb)							
			Analyzed		High	Low	Median	90%tile	95%tile	Mean§	SEM§	LOD†
Fluvalinate	PYR	117	140	83.6	5860.0	1.1	53.0	610.8	1710.0	357.7	94.5	1.0
Coumaphos	OP	84	140	60.0	762.0	1.0	8.0	118.7	156.2	50.4	13.5	1.0
Chlorpyrifos	OP	12	140	8.6	10.7	1.0	2.2	8.5	9.7	3.4	0.9	0.1
Chlorothalonil	FUNG	10	140	7.1	878.0	1.5	7.2	121.1	499.5	100.2	86.5	1.0
Cypermethrin	PYR	9	140	6.4	25.8	2.0	3.5	22.0	23.9	10.1	3.2	1.0
Permethrin	PYR	8	140	5.7	19600.0	12.0	35.8	5919.2	12759.6	2478.1	2446.0	10.0
DMPF (amitraz)	FORM	8	125	6.4	9040.0	6.0	117.5	3015.8	6027.9	1249.1	1114.1	4.0
Esfenvalerate	PYR	8	140	5.7	9.3	1.0	3.5	8.5	8.9	4.3	1.2	0.5
Methidathion	OP	7	140	5.0	32.0	6.5	12.0	28.4	30.2	16.2	3.6	1.0
Deltamethrin	PYR	6	140	4.3	39.0	23.0	26.5	38.5	38.8	29.3	3.0	20.0
Pendimethalin	HERB	6	140	4.3	27.6	6.5	14.0	26.4	27.0	15.9	3.8	1.0
Cyfluthrin	PYR	5	140	3.6	14.0	2.0	10.0	13.2	13.6	8.2	2.4	1.0
Dicofol	OC	5	140	3.6	3.8	1.0	1.4	3.6	3.7	2.1	0.6	0.4
Fenpropathrin	PYR	4	140	2.9	37.0	2.8	14.2	32.8	34.9	17.1	8.0	0.4
Azinphos methyl	OP	4	140	2.9	22.0	4.8	13.1	20.5	21.3	13.3	3.9	3.0
Cyprodinil	S FUNG	4	140	2.9	19.0	9.2	11.0	16.6	17.8	12.6	2.2	5.0
THPI (captan)	PS FUNG	3	125	2.4	43.4	37.7	39.5	42.6	43.0	40.2	1.7	30.0
Allethrin	PYR	3	140	2.1	24.0	6.7	19.0	23.0	23.5	16.6	5.1	1.0
Tetramethrin	PYR	3	140	2.1	23.0	18.0	23.0	23.0	23.0	21.3	1.7	6.0
Methoxyfenozide	IGR	3	140	2.1	21.0	1.5	3.4	17.5	19.2	8.6	6.2	0.4
Endosulfan I	CYC	3	140	2.1	6.1	1.3	1.6	5.2	5.7	3.0	1.6	0.1
Endosulfan sulfate	CYC	3	140	2.1	3.0	1.6	2.7	2.9	3.0	2.4	0.4	0.1
Endosulfan II	CYC	3	140	2.1	2.4	1.4	1.9	2.3	2.4	1.9	0.3	0.1
Parathion methyl	OP	3	140	2.1	2.0	1.5	1.8	2.0	2.0	1.8	0.1	1.0
Cyhalothrin	PYR	3	140	2.1	1.8	1.1	1.7	1.8	1.8	1.5	0.2	0.1
DMA (amitraz)	FORM	2	125	1.6	4740.0	275.0	2507.5	4293.5	4516.8	2507.5	2232.5	50.0
Fipronil	INS	2	140	1.4	3060.0	9.9	1535.0	2755.0	2907.5	1535.0	1525.1	1.0
Bifenthrin	PYR	2	140	1.4	12.3	2.9	7.6	11.4	11.8	7.6	4.7	0.4
Dieldrin	CYC	2	140	1.4	12.0	10.0	11.0	11.8	11.9	11.0	1.0	4.0
Prallethrin	PYR	2	140	1.4	8.6	6.2	7.4	8.4	8.5	7.4	1.2	4.0
Coumaphos oxon	OP	2	140	1.4	6.8	2.1	4.5	6.3	6.6	4.5	2.4	5.0
Oxyfluorfen	HERB	2	140	1.4	4.8	3.8	4.3	4.7	4.8	4.3	0.5	0.5
Chlorfenapyr	PS MITI	2	140	1.4	2.7	1.8	2.3	2.6	2.7	2.3	0.5	1.0
Carbaryl	PS CARB	1	140	0.7	588.0	588.0	588.0	588.0	588.0	588.0	---	5.0
1-Naphthol (carbaryl)	S CARB	1	140	0.7	238.0	238.0	238.0	238.0	238.0	238.0	---	2.0
Dimethomorph	S FUNG	1	125	0.8	56.0	56.0	56.0	56.0	56.0	56.0	---	15.0
Tebuconazole	S FUNG	1	140	0.7	34.0	34.0	34.0	34.0	34.0	34.0	---	3.0
Chlorferone (coumaphos)	OP	1	125	0.8	25.0	25.0	25.0	25.0	25.0	25.0	---	25.0
Tebufenozide	IGR	1	140	0.7	23.0	23.0	23.0	23.0	23.0	23.0	---	2.0
Fenoxaprop-ethyl	S HERB	1	140	0.7	15.4	15.4	15.4	15.4	15.4	15.4	---	6.0
Atrazine	S HERB	1	140	0.7	15.0	15.0	15.0	15.0	15.0	15.0	---	1.0
Carbendazim	S FUNG	1	140	0.7	14.3	14.3	14.3	14.3	14.3	14.3	---	1.0
Pyraclostrobin	FUNG	1	140	0.7	8.6	8.6	8.6	8.6	8.6	8.6	---	1.0
DDE p,p'	OC	1	140	0.7	6.6	6.6	6.6	6.6	6.6	6.6	---	3.0
Fluridone	S HERB	1	140	0.7	6.5	6.5	6.5	6.5	6.5	6.5	---	5.0
Pronamide	S HERB	1	140	0.7	2.2	2.2	2.2	2.2	2.2	2.2	---	1.0

*Carbendazim is also a degradate of benomyl; Thiabendazole is a degradate of thiophanate methyl.

# Class: CAR  =  carbamate, CYC  =  cyclodiene, FORM  =  formamidine, FUNG  =  fungicide, HERB  =  herbicide, IGR  =  insect growth regulator, INS  =  misc. insecticide, MITI  =  miticide, NEO  =  neonicotinoid, OC  =  organochlorine, OP  =  organophosphate, PS  =  partial systemic, PYR  =  pyrethroid, S  =  systemic.

§ Mean and SEM for detections > LOD.

† LOD  =  limit of detection (ppb).

Multiple residues prevailed in the bee, pollen and wax samples, with 2 or more pesticides detected in 92.3% of 749 analyzed (**Table 4**). Almost half of these samples (49.9%) contained at least one systemic pesticide. The most frequent binary pair of detections were the miticides fluvalinate and coumaphos found in 77.7% of samples, followed by the pyrethroid fluvalinate with the fungicide chlorothalonil (41.2%), fluvalinate with the organophosphate chlorpyrifos (39.4%), and the organophosphate coumaphos with chlorothalonil (39.1%). All 393 bee, pollen or wax samples with a fungicide detection (52.5%), except 9, had at least one other pyrethroid or organophosphate insecticide/miticide present. The most prevalent ternary combinations contained fluvalinate and coumaphos with chlorothalonil (38.6% of samples analyzed), chlorpyrifos (34.4%) or degradates of the miticide amitraz (32.6%). At least one each of an insecticide/acaricide, fungicide or herbicide were found in 28.5% of samples. The highest frequency of quaternary combinations of pesticides were the three miticides, fluvalinate, coumaphos and amitraz, with chlorothalonil (24%) or chlorpyrifos (15.7%) or fluvalinate, coumaphos, chlorothalonil and chlorpyrifos (19.2%).

**Table 4:** Pesticide incidence in 749 wax, pollen and bee samples from North American honey bee colonies.

Total Pesticide*	CLASS#	Samples Analyzed			% with Detections			LD50¶	Max Detection (ppb)			95%tile (ppb)			Wax (ppb) §		Pollen (ppb) §		Bee (ppb) §	
		Wax	Pollen	Bee	Wax	Pollen	Bee	(ppb)	Wax	Pollen	Bee	Wax	Pollen	Bee	Mean	SEM	Mean	SEM	Mean	SEM
Fluvalinate	PYR	259	350	140	98.1	88.3	83.6	15860	204000	2670	5860	28703	294	1623	7329.5	956.9	83.9	11.2	299.0	79.7
Coumaphos	OP	259	350	140	98.1	75.4	60.0	46300	94131	5828	762	11555	730	135	3363.4	511.8	137.4	25.4	30.5	8.4
Chlorpyrifos	OP	258	350	140	63.2	43.7	8.6	1220	890	830	11	33	127	1	15.5	4.8	23.3	4.8	0.3	0.1
Chlorothalonil	FUNG	258	280	140	49.2	52.9	7.1	1110000	53700	98900	878	1545	10380	3	525.0	225.2	1593.5	473.5	7.2	6.3
Amitraz	FORM	177	247	125	61.6	31.2	6.4	750000	46060	1117	13780	4700	181	6	1080.7	327.1	32.5	7.3	107.2	104.2
Pendimethalin	HERB	176	247	140	27.8	45.7	4.3	665000	84	1730	28	11	71	0	3.0	0.7	20.4	7.3	0.7	0.3
Endosulfan	CYC	258	350	140	39.1	36.6	3.6	78700	132	157	9	22	33	0	5.5	1.0	6.0	1.0	0.2	0.1
Fenpropathrin	PYR	258	350	140	17.1	18.0	2.9	500	200	170	37	30	12	0	4.2	1.0	2.7	0.7	0.5	0.3
Esfenvalerate	PYR	258	350	140	16.7	11.7	5.7	2240	56	60	9	11	3	1	1.5	0.3	0.9	0.3	0.2	0.1
Atrazine	S HERB	208	350	140	13.9	20.3	0.7	980000	31	49	15	8	17	0	1.1	0.3	2.8	0.4	0.1	0.1
Methoxyfenozide	IGR	208	350	140	18.8	8.3	2.1	1000000	495	128	21	89	11	0	15.3	3.9	2.9	0.8	0.2	0.2
Azoxystrobin	S FUNG	258	350	140	15.5	15.1	0.0	1120000	278	107	0	7	17	0	2.4	1.1	3.2	0.6	0.0	0.0
Bifenthrin	PYR	258	350	140	12.8	5.1	1.4	150	56	13	12	6	0	0	1.3	0.4	0.2	0.1	0.1	0.1
Trifluralin	HERB	176	247	125	12.5	3.6	0.0	685000	36	14	0	1	0	0	0.5	0.2	0.1	0.1	0.0	0.0
Aldicarb	S CARB	208	350	140	10.6	6.0	0.0	3730	693	1342	0	217	92	0	27.8	7.9	31.2	8.4	0.0	0.0
Carbendazim	S FUNG	208	350	140	10.1	4.6	0.7	500000	133	149	14	11	0	0	2.3	0.8	0.9	0.5	0.1	0.1
Boscalid	S FUNG	208	350	140	10.1	0.9	0.0	1550000	388	962	0	80	0	0	11.1	3.1	2.8	2.7	0.0	0.0
Dicofol	OC	258	350	140	10.1	8.0	3.6	370000	21	143	4	5	3	0	0.7	0.2	1.9	0.6	0.1	0.0
Iprodione	FUNG	208	350	140	6.7	0.3	0.0	1020000	636	10	0	136	0	0	18.2	5.8	0.0	0.0	0.0	0.0
Norflurazon	S HERB	208	350	140	6.3	5.1	0.0	1630000	38	108	0	2	2	0	0.4	0.2	1.5	0.5	0.0	0.0
Pyrethrins	PYR	208	350	140	6.3	0.9	0.0	1480	222	62	0	22	0	0	5.3	1.7	0.4	0.2	0.0	0.0
Oxyfluorfen	HERB	258	350	140	6.2	2.0	1.4	1000000	34	5	5	3	0	0	0.7	0.2	0.0	0.0	0.1	0.0
Methidathion	OP	258	350	140	5.8	4.0	5.0	2010	79	33	32	4	0	0	0.9	0.3	0.9	0.2	0.8	0.3
Fenbuconazole	S FUNG	176	247	125	5.7	5.7	0.0	1490000	183	264	0	11	0	0	3.1	1.3	4.6	1.6	0.0	0.0
Cyprodinil	S FUNG	208	350	140	5.3	4.3	2.9	3320000	106	344	19	4	0	0	1.8	0.8	3.9	1.6	0.4	0.2
Cyhalothrin	PYR	258	350	140	5.0	10.9	2.1	790	17	28	2	0	2	0	0.3	0.1	0.4	0.1	0.0	0.0
Cypermethrin	PYR	258	350	140	5.0	7.1	6.4	1350	131	49	26	1	3	2	1.6	0.7	0.8	0.2	0.6	0.3
Vinclozolin	FUNG	258	350	140	5.0	1.1	0.0	1000000	27	4	0	0	0	0	0.4	0.2	0.0	0.0	0.0	0.0
Tebufenozide	IGR	208	350	140	4.8	7.7	0.7	2340000	28	58	23	0	8	0	0.4	0.2	1.1	0.3	0.2	0.2
Pronamide	S HERB	208	350	140	4.8	3.1	0.7	1580000	23	378	2	0	0	0	0.3	0.1	3.9	1.6	0.0	0.0
Pyraclostrobin	FUNG	208	350	140	4.8	0.6	0.7	870000	438	265	9	0	0	0	4.0	2.3	0.8	0.8	0.1	0.1
Cyfluthrin	PYR	258	350	140	4.3	6.9	3.6	220	45	34	14	0	2	0	0.5	0.2	0.5	0.1	0.3	0.2
Allethrin	PYR	208	350	140	3.8	1.1	2.1	48800	139	11	24	0	0	0	1.1	0.7	0.1	0.0	0.4	0.2
Trifloxystrobin	PS FUNG	258	350	140	3.1	7.7	0.0	1750000	22	264	0	0	5	0	0.2	0.1	2.6	1.0	0.0	0.0
Deltamethrin	PYR	258	350	140	3.1	3.1	4.3	500	613	91	39	0	0	0	6.5	2.9	2.1	0.6	1.3	0.5
Azinphos methyl	OP	258	350	140	2.3	6.6	2.9	2420	121	643	22	0	10	0	0.8	0.5	5.7	2.6	0.4	0.2
Malathion	OP	258	350	140	2.3	3.7	0.0	3950	35	61	0	0	0	0	0.3	0.2	0.4	0.2	0.0	0.0
Phosmet	OP	258	350	140	1.9	5.7	0.0	8030	209	418	0	0	2	0	1.3	0.9	6.3	2.1	0.0	0.0
Permethrin	PYR	258	350	140	1.9	2.6	5.7	1120	372	92	19600	0	0	12	4.1	2.1	1.0	0.4	141.6	140.0
Diazinon	OP	208	350	140	1.9	7.7	0.0	2220	4	29	0	0	3	0	0.0	0.0	0.7	0.2	0.0	0.0
Thiacloprid	S NEO	208	350	140	1.9	5.4	0.0	252000	8	115	0	0	5	0	0.1	0.1	1.3	0.5	0.0	0.0
Pyrimethanil	FUNG	208	350	140	1.4	5.4	0.0	1000000	28	83	0	0	1	0	0.2	0.1	1.0	0.4	0.0	0.0
Tebuthiuron	S HERB	208	350	140	1.4	2.3	0.0	650000	22	48	0	0	0	0	0.2	0.1	0.4	0.2	0.0	0.0
Thiabendazole	S FUNG	208	350	140	1.4	2.3	0.0	500000	76	6	0	0	0	0	0.5	0.4	0.1	0.0	0.0	0.0
Fipronil	INS	208	350	140	1.4	0.3	1.4	50	36	29	3060	0	0	0	0.2	0.2	0.1	0.1	21.9	21.9
Dimethomorph	S FUNG	176	247	125	1.1	2.8	0.8	308000	133	166	56	0	0	0	1.1	0.8	1.3	0.7	0.4	0.4
Metribuzin	S HERB	208	350	140	1.0	5.1	0.0	567000	8	44	0	0	1	0	0.0	0.0	0.3	0.1	0.0	0.0
Propiconazole	S FUNG	208	350	140	1.0	4.0	0.0	625000	227	361	0	0	0	0	1.9	1.3	4.4	1.6	0.0	0.0
Imidacloprid	S NEO	208	350	140	1.0	2.9	0.0	280	14	912	0	0	0	0	0.1	0.1	3.1	1.0	0.0	0.0
Captan	PS FUNG	258	350	140	0.8	17.7	2.1	1080000	69	10000	43	0	326	0	0.4	0.3	76.3	29.6	0.9	0.5
Fenhexamid	FUNG	176	247	125	0.6	3.6	0.0	1580000	9	129	0	0	0	0	0.1	0.1	1.3	0.6	0.0	0.0
Carbaryl	PS CARB	208	350	140	0.5	11.4	0.7	10500	5	1050	826	0	45	0	0.0	0.0	13.8	4.7	5.9	5.9
Metolachlor	PS HERB	208	350	140	0.0	14.9	0.0	1260000	0	103	0	0	10	0	0.0	0.0	2.0	0.4	0.0	0.0
Myclobutanil	S FUNG	208	350	140	0.0	4.0	0.0	1870000	0	981	0	0	0	0	0.0	0.0	7.7	3.6	0.0	0.0
Simazine	S HERB	208	350	140	0.0	3.4	0.0	967000	0	54	0	0	0	0	0.0	0.0	0.8	0.3	0.0	0.0
Acetamiprid	S NEO	208	350	140	0.0	3.1	0.0	99000	0	134	0	0	0	0	0.0	0.0	1.9	0.7	0.0	0.0
Indoxacarb	INS	208	350	140	0.0	3.1	0.0	600000	0	330	0	0	0	0	0.0	0.0	3.7	1.3	0.0	0.0
Pyrethroids	PYR	259	350	140	98.8	92.6	86.4	----	204000	2670	19626	28706	307	1680	7354.5	956.6	93.2	11.3	444.6	159.3
Organophosphates	OP	259	350	140	99.6	92.6	62.9	----	94131	5828	762	11584	823	137	3383.1	511.7	175.2	25.7	32.0	8.4
Carbamates	CARB	208	350	140	13.0	18.0	0.7	----	693	1342	826	217	308	0	28.1	7.9	45.4	9.5	5.9	5.9
Insecticides	Total	259	350	140	99.6	98.9	91.4	----	213597	5832	19630	43006	1306	3190	11872.4	1131.2	370.0	30.7	612.4	187.1
Fungicides	FUNG	258	350	140	63.2	60.6	12.9	----	53705	98905	897	1578	3930	36	563.1	224.8	1388.1	381.2	9.2	6.4
Herbicides	HERB	208	350	140	41.8	50.3	6.4	----	560	1750	28	29	102	7	7.8	2.7	28.8	5.7	1.0	0.4
Systemics	S	208	350	140	57.7	60.9	10.7	----	723	1436	845	364	609	15	53.8	9.4	117.2	12.7	8.2	6.1
Pesticides	Total	259	350	140	99.6	99.1	91.4	----	213597	99936	19630	43492	7740	3219	12443.3	1145.5	1786.9	385.3	622.6	187.4

*Aldicarb based on sulfoxide and sulfone metabolites; Amitraz based on total DMA and DMPF metabolites; Captan includes THPI; Carbaryl includes 1-naphthol; Carbendazim is also a degradate of benomyl; Coumaphos includes oxon, chlorferone and potasan; Endosulfan includes isomers and sulfate; Imidacloprid includes 5-hydroxy and olefin metabolites; Thiabendazole is a degradate of thiophanate methyl.

# Class: CAR  =  carbamate, CYC  =  cyclodiene, FORM  =  formamidine, FUNG  =  fungicide, HERB  =  herbicide, IGR  =  insect growth regulator.

INS  =  misc. insecticide, NEO  =  neonicotinoid, OC  =  organochlorine, OP  =  organophosphate, PS  =  partial systemic, PYR  =  pyrethroid, S  =  systemic.

¶ LD50 is ave. honey bee acute toxicity from literature in ppb  =  10,000× µg/bee; bold nos. < LD50.

§ Mean and SEM for all analyzed samples; non detects  =  0 ppb.

Focused analysis of detections from only the pollen and wax further indicates the high potential of bee exposure to hive pesticide residues. Two or more pesticides were found in 98.4%, three or more in 91%, and four or more in 80% of the 609 samples analyzed. Almost 60% of these pollen and wax samples, in contrast to 10.7% of bee samples, contained at least one systemic pesticide, 57% in combination with a pyrethroid. The most frequent binary combination was fluvalinate and coumaphos (83.1% of samples), followed by fluvalinate with chlorothalonil (50.0%), coumaphos with chlorothalonil (47.8%), and fluvalinate with chlorpyrifos (46.7%). All 375 pollen or wax samples with a fungicide residue (61.7%) had at least one other insecticide or miticide present, and except for 6 or 8 of these samples, respectively, contained a pyrethroid or organophosphate. The most prevalent triple detections were fluvalinate and coumaphos combined with chlorothalonil (47.2%), chlorpyrifos (41.0%), degradates of amitraz (41.0%), or with one of 43 systemic pesticides (47.9%). At least one each of an insecticide/acaricide, fungicide or herbicide were found in 34.8% of samples, with the fluvalinate, chlorothalonil and pendimethalin combination most frequent (20.6%). The highest frequency of quaternary detections were fluvalinate, coumaphos and amitraz combined with chlorothalonil (30.7%) or chlorpyrifos (20.3%), or fluvalinate, coumaphos and chlorothalonil combined with a systemic (31.4%) or chlorpyrifos (26.2%).

### Trends in residue levels across the three primary matrices

The most frequently found residues were from fluvalinate and coumaphos, followed in order by chlorpyrifos, chlorothalonil, amitraz, pendimethalin, endosulfan, fenpropathrin, esfenvalerate and atrazine. These top ten comprise three in-hive miticides and five insecticidal, one fungicidal and one herbicidal crop protection agents (**Table 4**). In pollen, unprecedented levels (up to 99 ppm) of chlorothalonil were found, along with ppm levels of aldicarb, captan, carbaryl, myclobutanil, pendimethalin and the *Varroa* miticides (**Tables 2** **,** **4**). Near ppm levels of imidacloprid, boscalid and chlorpyrifos were also noted in pollen, with lesser but substantial amounts of potentially synergistic fungicides such as fenbuconazole, cyprodinil and propiconazole. Almost all wax samples (98%) were contaminated with fluvalinate and coumaphos up to 204 and 94 ppm, respectively, along with lower amounts and frequency of amitraz degradates and chlorothalonil. Near ppm levels of chlorpyrifos, aldicarb, deltamethrin, iprodione and methoxyfenozide were also found in comb wax (**Tables 1** **,** **4**).

Lower residues of pesticides prevailed in bees except for occasional samples associated with high mortality (see below) or with notable miticide (up to 14 ppm), and near ppm carbaryl and chlorothalonil detections (**Tables 3** **,** **4**). Although a few residues for atrazine, carbendazim, cyprodinil, pronamide, dimethomorph, and the degradates THPI (captan) and 1-naphthol (carbaryl) were detected, systemic pesticides were generally absent from bee samples (**Table 3**). No neonicotinoid residues were found in bees, while 23 thiacloprid, 14 imidacloprid, 11 acetamiprid and 1 thiamethoxam detections were obtained from pollen and wax (**Tables 1** **,** **2**, **3** **,** **4**). Overall, pyrethroids and organophosphates dominated total wax and bee residues followed by fungicides, systemics, carbamates and herbicides, whereas fungicides prevailed in pollen followed by organophosphates, systemics, pyrethroids, carbamates and herbicides (**Table 4**). The 98 pesticides and metabolites detected in mixtures up to 214 ppm in bee pollen alone represents a remarkably high level for toxic contaminants in the brood and adult food of this pollinator.

Pesticide residues ranged over six orders of magnitude (1 million-fold), and wide-differences in mean, and 90%- and 95%-tile values (levels at which only 10% or 5% of detections, respectively, are higher) per matrix were found (**Tables 1**, **2**, **3**, **4**). By comparing these residue levels across the matrices, an interesting trend emerges with regard to in-hive versus externally-derived pesticides. Fluvalinate, coumaphos and amitraz were 87-, 25- and 33-fold more concentrated in wax, respectively, than pollen (**Table 4**), while higher or more equivalent amounts of aldicarb, chlorothalonil, chlorpyrifos, endosulfan, pendimethalin, fenpropathrin, azoxystrobin and other environmental pesticides were found in the pollen compared to wax. This is consistent with chronic use and long-term accumulation of these lipophilic miticides in the wax, which becomes a source of subsequent contamination of stored pollen. For agricultural pesticides, the greatest indication of wax bioaccumulation from a pollen source is with the highly lipophilic insect growth regulator, methoxyfenozide, which was 5.3 times more prevalent in wax (**Table 4**). In general, this trend also occurred with the pyrethroids.

The highly-lipophilic fluvalinate and amitraz degradates (DMPF and DMA) bioaccumulate in bees to a much greater extent than does coumaphos, as indicated by the respective 3.6- and 3.3-times greater bee to pollen ratios of mean residue values relative to a 4.5-fold lower ratio for coumaphos (**Table 4**). The lipophilic fungicide chlorothalonil is 100-fold lower in bees than in pollen or wax, perhaps due to rapid bee transformation to undetected or excreted metabolites. Similar metabolism may explain the lower levels of coumaphos in bees compared to the other miticides. Parent fungicides and some metabolites (e.g THPI), regardless of lipophilicity or systemic movement, were generally lacking in bees, in contrast to being 151-times higher in pollen (**Table 4**).

### Pesticide residue levels and acute bee toxicity

Comparison of ppb residue levels across matrices with known LD_50_ values for honey bees in ppb relative to body weight provided only a few detections at or well above the lethal dose (**Table 4**). Two samples of dead bees were linked by analysis to prior environmental applications of permethrin (19.6 ppm residue, LD_50_ of 1.1 ppm) and fipronil (3.1 ppm, LD_50_ 0.05 ppm). However, other bee samples represented bees remaining, and it should be noted that foragers that never returned and were presumed dead were not sampled. For bees from CCD–associated colonies, only sublethal although high amounts of fluvalinate (up to 6 ppm), amitraz, coumaphos and chlorothalonil were detected. That the bee content for the latter lipophilic fungicide was much less (221 times on average) than beebread food (**Table 4**) from the same colonies indicates that metabolism of the parent pesticide is occurring in the bee. Detected pollen levels of pesticides are predicted to be sublethal (below one-tenth the LD_50_) except for occasional high residues of the pyrethroids cyfluthrin, deltamethrin, fenpropathrin, and fluvalinate; organophosphates azinphosmethyl, chlorpyrifos and coumaphos; carbamates aldicarb and carbaryl; and fipronil and imidacloprid (**Table 4**), depending on bee consumption rates. Wax residues are similarly expected to be sublethal, depending on transfer rates to brood or indirectly to food, except for occasional high levels of aldicarb, bifenthrin, chlorpyrifos, coumaphos, cyfluthrin, cypermethrin, deltamethrin, fenpropathrin, fipronil, fluvalinate, permethrin, and pyrethrins. The biological impacts of combinations of these materials at their dietary levels on either honey bee larvae or adults remains to be determined.

### In-hive comparisons of pesticide detections

**Figure 1:**
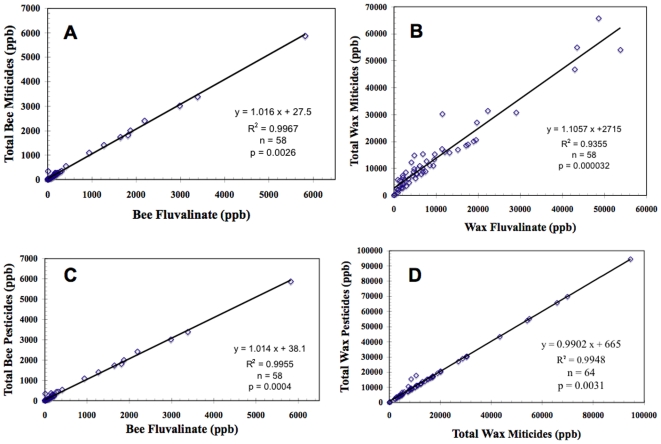
Correlations of bee and wax fluvalinate residues (ppb) with total miticide and pesticide contents in paired colony samples. Regressions of bee fluvalinate with total miticides (A), wax fluvalinate with total miticides (B), bee fluvalinate with total pesticides (C), and of wax miticides with total pesticides (D).

Pairing by colony/matrix for concurrently-sampled matrices, reduced our database to 519 analyses that averaged 6.5 detections per sample representing 102 different pesticides and metabolites. Colony analyses were then averaged according to matrix if sampling dates were not identical. The following significant trends were extracted by correlation followed by linear regression analysis of these data. Fluvalinate accounts for most of the miticide content of bees (bee miticide = 1.016 • bee fluvalinate +27.5 ppb; r^2^ = 0.9967, *p* = 0.0026, n = 58; **Fig. 1a**) and comb wax (wax miticide = 1.106 • wax fluvalinate +2715 ppb; r^2^ = 0.9355, *p* = 0.000032, n = 58; **Fig. 1b**). Fluvalinate explains most of the pesticide residues detected in bees (bee pesticides = 1.014 • bee fluvalinate +38.1 ppb; r^2^ = 0.9955, *p* = 0.0004, n = 58; **Fig. 1c**). Wax content is a much better correlative of bee levels of fluvalinate (wax fluvalinate = 8.53 • bee fluvalinate +5911 ppb; r^2^ = 0.522, *p* = 0.00001, n = 58) than the beebread (bee fluvalinate = 4.1 • pollen fluvalinate - 77 ppb; r^2^ = 0.366, *p* = 0.515, n = 41), consistent with wax being the primary source of bee contamination. Wax is also the primary source of the much lower bee residues of the other major hive miticide, coumaphos, as indicated by the highly significant correlation of wax and bee contents (wax coumaphos = 54.2 • bee coumaphos +1383 ppb; r^2^ = 0.484, *p* = 0.0015, n = 58) compared to the non-significant correlation of pollen and bee residues (r^2^ = 0.00585, *p* = 0.630, n = 42). Bee residues of the third miticide, amitraz, were not significantly related to either wax (r^2^ = 0.042) or pollen (r^2^ = 0.0036) contents. However, these three miticides accounted for the majority of pesticide residues in comb wax (wax pesticides = 0.9902 • wax miticides +665 ppb; r^2^ = 0.9948, *p* = 0.0031, n = 64; **Fig. 1d**).

**Figure 2:**
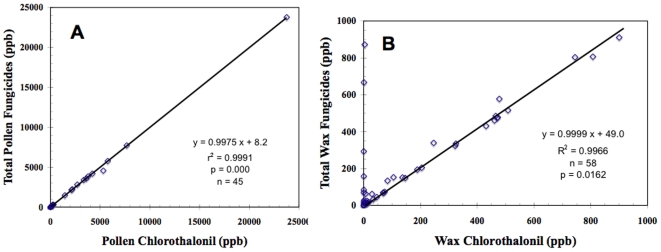
Correlations of pollen and wax chlorothalonil residues (ppb) with total fungicide contents in paired colony samples. Regressions of pollen chlorothalonil (A) and wax chlorothalonil (B) with total fungicides.

**Figure 3:**
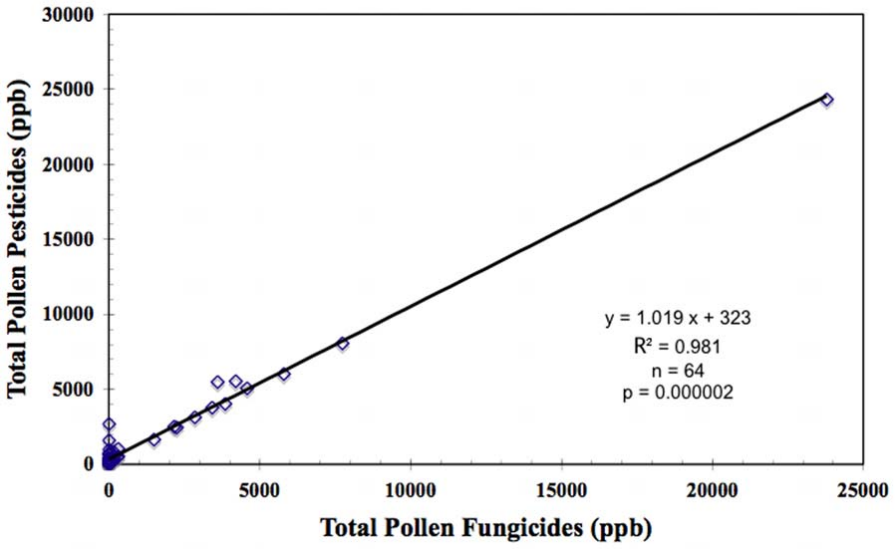
Correlation of total fungicide residues (ppb) with total pesticide contents of pollen samples.

Noteworthy trends uncovered here for pollen pesticide residues resulted from their high fungicide content. Most fungicide contents in bee-collected pollen were due to chlorothalonil (pollen fungicides = 0.9975 • pollen chlorothalonil +8.2 ppb; r^2^ = 0.9991, *p* = 0.000, n = 45; **Fig. 2a**) as were the fungicide residues of comb wax (wax fungicides = 0.9999 • wax chlorothalonil +49 ppb; r^2^ = 0.9966, *p* = 0.0162, n = 58; **Fig. 2b**). Indeed, fungicides accounted for most of the pesticide content of pollen (pollen pesticides = 1.019 • pollen fungicides +323 ppb; r^2^ = 0.981, *p* = 0.000002, n = 64; **Fig. 3**). In pollen, the non-systemic chlorothalonil also tended to co-occur with lower levels of systemic pesticides including particularly fungicides (pollen chlorothalonil = 45.6 • pollen systemics - 491 ppb; r^2^ = 0.8095, *p* = 0.10, n = 45).

Slopes from linear regression analyses, although with high variance, are consistent with pollen being the probable source of wax chlorothalonil (wax chlorothalonil = 0.502 • pollen chlorothalonil +79 ppb; r^2^ = 0.385, *p* = 0.70, n = 44), while pollen content of amitraz (wax amitraz = 33.2 • pollen amitraz +0.0 ppb; r^2^ = 0.800, *p* = N.A., n = 64), coumaphos (wax coumaphos = 5.3 • pollen coumaphos +1846 ppb; r^2^ = 0.569, *p* = 0.184, n = 63), and fluvalinate (wax fluvalinate = 2670 • pollen fluvalinate +6903 ppb; r^2^ = 0.0081, *p* = 0.48, n = 63) come from respective miticide residues in comb wax. The weak but significant correlation of greater levels of fluvalinate coincident with high coumaphos in the comb wax of colonies (wax fluvalinate = 1.406 • wax coumaphos +5586 ppb; r^2^ = 0.186, *p* = 0.004, n = 58), is consistent with frequent co-treatments with these miticides over the course of the colony year or life.

### Foundation wax is uniformly contaminated with miticides

Twenty-one wax samples from six different commercial and two private foundation sources were uniformly contaminated with up to 10.1 ppm fluvalinate (mean of 2±0.6 ppm) and up to 14.3 ppm coumaphos (mean of 3.3±1.0 ppm, **Table 5**), which is 27% and 100%, respectively, of mean detection levels found in comb wax overall (**Table 1**). One organic beekeeper source lacked coumaphos in its foundation, although 0.5 ppm of fluvalinate was still present. Much lower levels of 25 other pesticides and metabolites were found in 21 samples, at an average of 5.7 detections per sample, which is lower than the 8 detections per sample of comb wax overall. Systemics were found less often in foundation (5.8% of detections, **Table 5**) than in comb wax (**Table 1**). Other frequently detected contaminants include chlorpyrifos (81%), endosulfan (38%), chlorothalonil (29%) and other pyrethroids including cypermethrin, cyfluthrin and esfenvalerate (**Table 5**). Interestingly, three distinct old foundation samples from prior to miticide use lacked fluvalinate and coumaphos as expected, but contained more chlorpyrifos and significant levels of other pesticides no longer registered including bendiocarb, *p,p'*-DDE, and heptachlor (not shown).

**Table 5:** Summary of pesticide detections in foundation samples from North American honey bee colonies.

Pesticide*	Class#	Detects	Samples	%	Detections (ppb)						
			Analyzed		High	Low	Median	90%tile	95%tile	Mean§	SEM§
Fluvalinate	PYR	21	21	100.0	10120.0	2.0	455.0	6020.0	9810.0	2006.1	661.4
Coumaphos	OP	20	21	95.2	14300.0	1.0	1350.0	8867.0	12875.0	3315.1	962.7
Chlorpyrifos	OP	17	21	81.0	110.0	1.4	10.0	51.8	76.4	22.2	7.1
Endosulfan I	CYC	8	21	38.1	11.0	1.2	2.4	5.8	8.4	3.3	1.1
Coumaphos oxon	OP	7	13	53.8	102.0	6.5	27.3	62.4	82.2	36.0	11.6
Chlorothalonil	FUNG	6	21	28.6	60.0	1.3	11.4	39.1	49.6	18.2	8.7
Cypermethrin	PYR	5	21	23.8	131.0	6.5	8.3	120.2	125.6	51.6	27.3
Endosulfan II	CYC	5	21	23.8	4.7	1.1	1.9	3.6	4.1	2.1	0.7
Cyfluthrin	PYR	4	21	19.0	14.0	6.9	7.4	12.1	13.1	8.9	1.7
Esfenvalerate	PYR	4	21	19.0	19.0	1.1	2.8	14.2	16.6	6.4	4.2
Pendimethalin	HERB	2	11	18.2	7.2	7.1	7.2	7.2	7.2	7.2	0.0
Trifluralin	HERB	2	11	18.2	36.0	2.2	19.1	32.6	34.3	19.1	16.9
Allethrin	PYR	2	13	15.4	139.0	9.5	74.3	126.1	132.5	74.3	64.8
Fluridone	S HERB	2	13	15.4	6.6	5.7	6.2	6.5	6.6	6.2	0.4
Vinclozolin	FUNG	2	21	9.5	1.7	1.2	1.5	1.7	1.7	1.5	0.3
*p*-Dichlorobenzene	OC	1	3	33.3	6.9	6.9	6.9	6.9	6.9	6.9	---
Atrazine	S HERB	1	13	7.7	31.0	31.0	31.0	31.0	31.0	31.0	---
Norflurazon	S HERB	1	13	7.7	38.1	38.1	38.1	38.1	38.1	38.1	---
Parathion methyl	OP	1	13	7.7	3.8	3.8	3.8	3.8	3.8	3.8	---
Tebuthiuron	S HERB	1	13	7.7	5.8	5.8	5.8	5.8	5.8	5.8	---
Thiabendazole	S FUNG	1	13	7.7	76.0	76.0	76.0	76.0	76.0	76.0	---
Dicofol	OC	1	21	4.8	2.3	2.3	2.3	2.3	2.3	2.3	---
Endosulfan sulfate	CYC	1	21	4.8	1.6	1.6	1.6	1.6	1.6	1.6	---
Fenpropathrin	PYR	1	21	4.8	25.0	25.0	25.0	25.0	25.0	25.0	---
Methidathion	OP	1	21	4.8	14.0	14.0	14.0	14.0	14.0	14.0	---
Phosmet	OP	1	21	4.8	2.9	2.9	2.9	2.9	2.9	2.9	---
Trifloxystrobin	PS FUNG	1	21	4.8	3.5	3.5	3.5	3.5	3.5	3.5	---

*Thiabendazole is a degradate of thiophanate methyl.

# Class: CAR  =  carbamate, CYC  =  cyclodiene, FORM  =  formamidine, FUNG  =  fungicide, HERB  =  herbicide, IGR  =  insect growth regulator, INS  =  misc. insecticide, MITI  =  miticide, NEO  =  neonicotinoid, OC  =  organochlorine, OP  =  organophosphate, PS  =  partial systemic, PYR  =  pyrethroid, S  =  systemic.

§ Mean and SEM for detections > LOD.

† LOD  =  limit of detection (ppb).

### Pesticide degradates differ among matrices

Substantial levels of coumaphos oxon, the toxiPlos finished live paper--pone.0009730 oxidative metabolite of coumaphos, and the related degradate, chlorferone were frequently detected in comb wax (**Table 1**) compared to pollen (**Table 2**) or bees (**Table 3**). Coumaphos oxon (up to 1.3 ppm), which is the cytochrome P450-activated form of this acetylcholinesterase inhibitor [30], and chlorferone (up to 4.4 ppm), the phenolic hydrolysis product which is a highly photoreactive coumarin [31], were prevalent in wax, though the latter was absent from pollen samples and only detected once in bees. By contrast, the toxic, dechlorinated coumaphos metabolite, potasan, was absent from wax, but detected 3 times at up to 160 ppb in pollen. Both amitraz degradates DMPF (up to 43 ppm) and DMA (up to 3.8 ppm) prevailed in wax (**Table 1**) and to a lesser extent (up to 9 and 4.7 ppm, respectively) in bees (**Table 3**), whereas DMA was never detected in pollen even though its precursor DMPF occurred at up to 1.1 ppm (**Table 2**). Much higher amounts of the more bee-toxic aldicarb sulfoxide (up to 1.25 ppm) than its sulfone (up to 0.097 ppm) were frequently detected in pollen and wax samples, while both of these systemic metabolites were absent from bees. THPI, a systemic degradate of captan, and 1-naphthol, a systemic degradate of carbaryl, were never detected in wax (**Table 1**), although found 53 times in pollen and 4 times in bees (**Tables 2** **,** **3**). Thus, parent pesticide biotransformations to metabolites which are equally or more toxic than their parent compounds differs among matrices of the hive.

### High diversity of pesticides detected in beehive samples

We found 121 different pesticides and metabolites comprising 5519 total residues within 887 wax, pollen, bee and associated hive samples (average of 6.2 detections per sample) from 23 states and one Canadian province (**Table S1**). These included 16 parent pyrethroids, 16 organophosphates (13 parents, 3 metabolites), 8 carbamates (4 parents, 4 metabolites), 6 neonicotinoids (4 parents, 2 metabolites), 6 chlorinated cyclodienes (3 parents, 3 metabolites), 5 organochlorines (3 parents, 2 metabolite), 4 insect growth regulators, 2 formamidines (2 metabolites), 9 miscellaneous miticides/insecticides (8 parents, 1 metabolite), 2 synergists, 30 fungicides, and 17 herbicides. Of these detected pesticides and metabolites, 47 are systemic (**Table S1**). Among these compounds, 14 (12%) were detected only once, 20 (17%) twice, but 79 (65%) of these pesticides occurred in 6 or more samples, and 37 (31%) were found over 30 times. Pyrethroids were quantitatively the most prevalent of pollen residues with up to ten different parent compounds per sample. Among the 81 compounds analyzed for but not detected in these samples (**Table S2**), many are pesticides that degrade rapidly (e.g. aldicarb, amitraz), metabolites (15%), compounds infrequently used around bees (e.g. hydroprene), or chemicals cancelled for use (e.g. aldrin, endrin). There were no remarkable differences in trends reported between the focused database above and our complete database that includes a higher diversity of matrices except for 3 additional pesticides detected; indeed most extreme detections were from the wax, pollen and bee database of 749 samples.

## Discussion

We have found unprecedented levels of miticides and agricultural pesticides in honey bee colonies from across the US and one Canadian province. While these samples were not part of a full-scale landscape or grower-level survey, the data contained here is the largest sampling of pesticide residues in N. American bee colonies or worldwide to date, and represents a cost of nearly $175,000 for the analyses alone. We attempt here to draw trends from these data to indicate both potential risks for bee health as well as justifying the need for greater investments in monitoring pesticide residues in the future.

While a slightly larger number of pesticides are found by including materials associated with beekeeping such as corn syrup, pollen substitute, royal jelly, honey and floral nectars, the trends are well represented by the hive contents of pollen, wax, and bees. A comparison of **Tables 1** **,** **2** **,** **3** **,** **4** with **Tables** **S1** and **S2** indicates that a number of currently used pesticides (e.g. alachlor, dimethoate) were not found in any samples, and that some of the most environmentally persistent pesticides banned from use in the last 10 years (e.g. aldrin, endrin) also do not appear.

### High levels of multiple pesticides in bee-collected pollen

High levels of fluvalinate and coumaphos are co-occuring with lower but significant levels of 98 other insecticides, fungicides and herbicides in pollen. Most noteworthy were the very high levels of the fungicide chlorothalonil in pollen and wax (**Tables 1** **,** **2** **,** **4**) as well as ppm levels of the insecticides aldicarb, carbaryl, chlorpyrifos and imidacloprid, fungicides boscalid, captan and myclobutanil, and herbicide pendimethalin. With an average of 7 pesticides in a pollen sample, the potential for multiple pesticide interactions affecting bee health seems likely. Ten pesticides were found in pollen at greater than one tenth the bee LD_50_ level indicating that sublethal effects of these toxicants alone are highly likely. European researchers have noted fewer and usually lower levels of pesticides in pollen samples, although high detections of particularly carbamates and pyrethroids have been reported [8], [32].

As pollen is the main protein source for developing brood and is intimately involved in development of the hypopharyngeal glands of nurse bees [33], which in turn affects their ability to rear brood, surviving on pollen with an average of 7 different pesticides seems likely to have consequences. Requirements for protein at the colony level vary markedly over the growing season, and the ability of the hive as a superorganism to respond to these changing needs may be compromised by the plethora of pesticides we documented in pollen. Given the critical role played by pollen in bee nutrition and colony dynamics, the complete lack of understanding of chemical biotransformations of pesticides in stored beebread compels a need for additional work.

It is well documented that neonicotinoid pesticides occur in pollen at levels that affect the learning ability of bees fed such pollen [8]–[11], but adding other fungicides or pesticides into this mix has yet to be considered. Bees have genes for specific types of nicotinic acetylcholine receptors [34], and therein may lie the special sensitivity they have to neonicotinoids, but behavioral outcomes of selective actions at these molecular targets has yet to be investigated.

Growers of many bee-pollinated crops routinely apply fungicides during bloom, while pollinators are present [35] as there are currently no label restrictions for this action. Thus it may not be surprising that fungicides account for most of the pesticide content of pollen (**Figure 2a** **).** Kubik et al. [36] noted high residues of the fungicides vinclozolin and iprodione up to 32 and 5.5 ppm respectively, in beebread. Chlorothalonil is the most frequent detection in pollen and wax after fluvalinate and coumaphos, and all three coincide in 47% of our pollen and wax samples. Chlorothalonil is a highly reactive, widely-used, broad-spectrum fungicide that promotes oxygen stress [37] and is overtly toxic to fish and other aquatics at ppb levels [38]. We found chlorothalonil to be a marker for entombing behavior in bee colonies associated with poor health [24], and it was suggested that entombing may be a new defensive behavior of bees faced with large amounts of potentially toxic food stores. Pollen appears to be the source of chlorothalonil residues in wax, as the pollen levels are higher and correlative of the levels in wax from the same colonies (**Figure 2b** **)**. Chlorothalonil content in beebread is expectedly driven by bees foraging on this non-systemic fungicide either directly by picking up pollen-sized particle formulations or through their presence where pollen, nectar, or water is collected. Some fungicides have shown direct toxicity to honey or solitary bees at field use rates [39], but consequences of chlorothalonil in pollen and beebread fed to bee brood and adults alone or in conjunction with other pesticides remains to be determined.

### High levels of miticides in comb wax

Beeswax remains the ultimate sink from the long-term use of the miticides fluvalinate, coumaphos, amitraz (**Table 4**) and bromopropylate [40], reaching 204, 94, 46 and 135 ppm, respectively. Colony residue levels of these miticides, after their in-hive application, have been shown to increase from honey to pollen to beeswax [16], [40]–[45]. Beeswax is the resource of the hive that is least renewable and is thus where persistent pesticides can provide a “toxic-house” syndrome for the bees. The uniform high levels of these miticides present in foundation (**Table 5**) is particularly disturbing, since replacement of comb is currently recommended to reduce pesticide contaminants. The broad contamination of European foundation with especially miticides has been reviewed previously [43]. Fluvalinate residues in beeswax best correlated with the French bee winter kill of 1999–2000 [5], although disease factors were more emphasized in the report. Out of the surveyed apiaries suffering severe bee mortality, 79% of their wax samples contained this miticide in contrast to 76% harboring one or more serious diseases.

Almost all wax and pollen samples (98.4%) contained two or more pesticide residues, of which greater than 83% were fluvalinate and coumaphos (**Table 4**). Clearly, substantial residues of these bee-toxic pyrethroid and organophosphate compounds prevailed together in most beehives sampled. Chronic exposures to high levels of these persistent neurotoxicants elicits both acute and sublethal reductions in honey bee fitness, especially queens [46], [47], and they can interact synergistically on bee mortality [48]. Our work does not directly associate these miticides with CCD, although higher coumaphos levels may actually benefit the colony, possibly via mite control [18].

Almost 60% of our pollen and wax samples, in contrast to 11% of bee samples, contained at least one of 43 systemic pesticides, 57% in combination with a pyrethroid. Substantial amounts of potentially synergistic fungicides such as cyprodinil, fenbuconazole, myclobutanil and propiconazole were also found. Fungicides generally have low bee toxicity by themselves, but exceptions with captan and the ergosterol biosynthesis inhibitor (EBI) propiconazole have been reported [39]. The latter as well as myclobutanil are potent synergists for the pyrethroid cyhalothrin [49]. The frequent coincidence in pollen of high levels of the non-systemic fungicide chlorothalonil with lower levels of systemic pesticides including fungicides is another probable synergistic combination that needs further exploration concerning bee decline.

### Lower levels of pesticides in bees

Bees generally have lower pesticide residues than pollen [**Table 4**, 32]. Samples taken from unhealthy CCD-associated colonies were from live bees at the time of collection and represent house bees or residual foragers. These most likely were newly emerged bees, since older bees are typically missing from fully collapsed hives. Fluvalinate exceeded coumaphos residues in these bees, but even the highest detection of 6 ppm (**Table 4**) is less than half the LD_50_, and by itself could account for only a low death level. We found chlorothalonil at 100-fold lower concentrations in bees compared to pollen or wax indicating its rapid biotransformation to undetected or excreted metabolites (**Table 4**). Biotransformations and rapid excretion may also explain the general lack of systemic pesticide residues in bees.

### Broader trends of pesticides from associated hive matrices

Externally-derived, highly-toxic pyrethroids, up to 9 in addition to fluvalinate per sample, were the most frequent and dominant class of insecticides in our samples. Pyrethroids are frequently associated with bee kills [50]. A sample of dead bees, obtained after a community-wide tree application of permethrin according to label instructions, contained 19.6 ppm, 18-times the established bee LD_50_ (**Table 4**). Pollen and wax levels of more toxic pyrethroids including bifenthrin, cyfluthrin, cyhalothrin, deltamethrin, and fenpropathrin ranged up to 613 ppb, which is above the bee LD_50_ for deltamethrin. This level can be lethal depending on pollen consumption rates by differing castes, or wax transfer rates to brood or indirectly to pollen. Moreover, some bee residues of deltamethrin, fenpropathrin and cypermethrin (**Table 4**) are above levels shown to disorient foragers [51] and cause CCD-like symptoms (see above). It is important to note that pyrethroids are rarely found alone, and in 50% of our pollen and wax samples co-occur with chlorothalonil, a fungicide known to increase bee toxicity of cypermethrin by greater than 5-fold [52]. Bee toxicity of the pyrethroid bifenthrin doubles after Apistan (fluvalinate) treatment [53], which frequently coincides in our samples. Potential for interactions among multiple pyrethroids and fungicides seems highly likely to impact bee health in ways yet to be determined.

Pyrethroids other than fluvalinate have been reported to impact the foraging capabilities of honey bees. After topical application with 0.009 µg permethrin/bee (approx. 90 ppb body weight), none of the foraging workers returned to the hive at days end [54], and only 43% of these bees returned even once to the hive because of disorientation due to the treatment. vanDame et al. [51] found a similar effect on foragers with deltamethrin at 0.0025 µg/bee (25 ppb), a dose 27 time lower than the LD_50_, which disoriented 91% of return bee flights to the hive. These symptoms are reminiscent of those reported for CCD.

Other classes of pesticides have been associated with bee kills including 3.1 ppm of the phenylpyrazole fipronil (**Table 4**). Anderson and Wojtas [55] linked dead honey bees to high residues of the carbamates carbaryl (5.8 ppm) and methomyl (3.4 ppm), cyclodienes chlordane (0.7 ppm) and endosulfan (4.4 ppm), organophosphates malathion (4.2 ppm) and methyl parathion (3.6 ppm), and the fungicide captan (1.7 ppm). Walorczyk and Gnusowski [56] found exceptional amounts of the organophosphates dimethoate (4.9 ppm), fenitrothion (1 ppm), and omethoate (1.2 ppm), and up to 1.2 ppm of the systemic fungicide tebuconazole in bees from other poisoning incidences. Similarly, elevated residues of the organophosphates bromophos methyl (1.7 ppm) and fenitrothion (10.3 ppm) were associated with high bee mortality [57].

Pesticide metabolites (enzymatically-produced) and degradates (chemically-produced or of unknown origin) can be as toxic and are often more systemic than their respective parent compounds. Systemic movement can enhance their levels in floral pollens and nectars, but their increased water solubility can also facilitate excretion from bees. Much higher amounts of the more bee-toxic aldicarb sulfoxide than its sulfone [58] were frequently detected in pollen samples from hives near citrus, while both of these systemic metabolites were absent from bees. The systemic degradates THPI from captan and 1-naphthol from carbaryl were often found in pollen but much less frequently in bees. Parent pesticide biotransformations to metabolites differs among the bees, their food pollens and wax comb. Thus, complications for bee health may result from pesticide metabolism in hive and foraging sites to more systemic or otherwise water-soluble metabolites which are equally or more toxic than their parent compounds. Once again, data for combinations of these metabolites with parent compounds in mixtures of two or more components are completely lacking in the literature.

The affects of chronic exposure to pyrethroids, organophosphates, neonicotinoids, fungicides and other pesticides can range from lethal and/or sub-lethal effects in brood and workers to reproductive effects on the queen [59]. Bee nutrition and physiological changes across seasons (summer versus winter bees) can have marked impacts on their pesticide susceptibility [60]. Attempts to correlate global bee declines or CCD with increased pesticide exposures alone [18], [32] have not been successful to date. Two major complications with such attempts are that the time delay between collecting pollen contaminated with multiple pesticides, as we have shown here, and when it is actually consumed by bees or brood is not predictable in colonies, and the potential biotransformations of pesticides in beebread are completely undocumented. Pesticide interactions among various mixures as well as with other stressors including *Varroa* and *Nosema* [18], IAPV [17], beneficial hive microbes [61], [62], and impacts on bee immune systems all require further study. It seems to us that it is far too early to attempt to link or to dismiss pesticide impacts with CCD.

### Implications for bee research on the roles of pesticides in bee health

Systemic neonicotinoid use has greatly increased recently for treating seeds of many major crops, particularly those genetically-engineered [9], [63], [64], and considerable impact to non-target species may occur [65]. Neonicotinoids and systemic fungicides are often combined as pest control inputs, and many of the latter synergize the already high bee toxicity of neonicotinoids [66]. Bee kills in France and Germany have been associated with particularly imidacloprid [9] and clothianidin [67]. Although a few residues for atrazine, carbendazim, cyprodinil, pronamide, dimethomorph, and the degradates THPI (captan) and 1-naphthol (carbaryl) were detected, systemic pesticides were generally absent from bee samples (**Table 3**). No neonicotinoid residues were found in bees, while 49 detections were obtained from pollen and wax (**Tables 1** **,** **2** **,** **3** **,** **4**). Our results do not support sufficient amounts and frequency in pollen of imidacloprid (mean of 3.1 ppb in less than 3% of pollen samples) or the less toxic neonicotinoids thiacloprid and acetamiprid to account for impacts on bee health, although one pollen sample contained an exceptional level of 912 ppb imidacloprid (**Table 4**). A recent landscape-level study of imidacloprid seed treatments on maize in Belgium demonstrated no impacts on honey bees [68]; however, their high prevalence with EBI and other fungicides [49], [66] including myclobutanil [16], although refuted by some field results [69], may have more direct impacts on bee health through synergistic combinations.

The high frequency of multiple pesticides in bee collected pollen and wax indicates that pesticide interactions need thorough investigation before their roles in decreasing bee health can be either supported or refuted. The large number of studies to date, are limited by being done on mostly one compound at a time, as well as using whole colonies where the timing of contaminated pollen intake and its utilization by the colony are difficult to interpret as a causal relationship. Laboratory studies have clearly indicated sublethal impacts on honey bee learning [10], immune system functioning [11], and synergism of insecticide toxicity by fungicides, yet combinations of herbicides with fungicides and insecticides in 3 or more component mixtures have not been studied. Seasonal and genetic changes in bee sensitivity to pesticides [60] and nutritional levels [33] are known, but again the interactions of these with the above combinations of chemicals remain to be determined.

### Implications for colony management to minimize pesticide impacts

Fluvalinate has been considered a relatively “safe” material for honey bees by the beekeeping industry; however its history is unclear with potentially significant implications for honey bee health. The original formulation of fluvalinate had an established lethal dose that killed 50% of the tested population (LD_50_) of 65.85 µg/bee for honey bees, which is considered relatively non-toxic [27]. Surprisingly, EPA in 1995 reported the LD_50_ of fluvalinate as 0.2 µg/bee, a level that is considered to be highly toxic [70] to honey bees. This is 330-fold more toxic than indicated by the original LD_50_, a value still quoted in current literature [e.g. 8]. Extraordinary enhancement of toxicity has been found with addition of commercial synergists to fluvalinate, where a topical LD_50_ of 0.00964 µg/bee, a 980-fold increase to their reported 9.45 µg/bee without the additive, occurred if 100 µg of piperonyl butoxide was applied 1 hr prior to the pyrethroid [71]. Centrally-acting neurotoxicants can sublethally impact a social bee more than the targeted pest due to the complex communication and sensory-based behaviors required to maintain community organization.

Widely-occurring *Varroa* mite resistance to fluvalinate, coumaphos and now amitraz may have developed rapidly as a result of their constant exposure to miticide-impregnated wax comb. Removal of these residues from wax may extend the usefulness of these or future miticides, by reducing this high selection pressure. It is generally agreed that the mite, *Varroa destructor* Anderson & Trueman, is playing a key role in the demise of honey bee health, and that intensive use of miticides for their control has led to evolution of wide-spread mite resistance among European strains of honey bees [72]–[75]. Fluvalinate and coumaphos, but not amitraz, are highly persistent in the hive with an estimated half-life in beeswax of 5 years [43]. Fortunately, a broad sampling of U.S. honey showed frequent but very low levels of coumaphos and fluvalinate up to 12 ppb, and only a few detections of lesser amounts of four other pesticides [76].

### Implications for regulatory policy to minimize pesticide risks for pollinators

The widespread occurrence of multiple residues, some at toxic levels for single compounds, and the lack of any scientific literature on the biological consequences of combinations of pesticides, argues strongly for urgent changes in regulatory policies regarding pesticide registration and monitoring procedures as they relate to pollinator safety. This further calls for emergency funding to address the myriad holes in our scientific understanding of pesticide consequences for pollinators. The relegation of bee toxicity for registered compounds to impact only label warnings, and the underestimation of systemic pesticide hazards to bees in the registration process may well have contributed to widespread pesticide contamination of pollen, the primary food source of our major pollinator. Is risking the $14 billion contribution of pollinators to our food system really worth lack of action?

## Supporting Information

Table S1 Summary of pesticide detections in 887 North American beehive and related samples.
(0.37 MB DOC)

Table S2 Summary of pesticides and their metabolites not detected in 887 North American beehive and related samples.
(0.20 MB DOC)
