# Supplementary material for: High Levels of Miticides and Agrochemicals in North American Apiaries: Implications for Honey Bee Health
Source: PLoS One. 2010 Mar 19;5(3):e9754. doi: 10.1371/journal.pone.0009754 (PMC2841636; doi:10.1371/journal.pone.0009754)
Supplement: Table S1 — Summary of pesticide detections in 887 North American beehive and related samples. (0.37 MB DOC) [file pone.0009754.s001.doc]

**Table S1.** Summary of pesticide detections in 887 North American beehive and related samples.

| **Pesticide or Metabolite** | **Class^#^** | **LOD^†^** | **Samples Analyzed** | **Total Detections** | **% of Samples** | **Mean^§^ (ppb)** | **SEM^§^ (ppb)** | **Lowest Value** | **Highest Value** | **LD50^¶^ (ppb)** |
| --- | --- | --- | --- | --- | --- | --- | --- | --- | --- | --- |
| 1-Naphthol (carbaryl) | S CARB | 2 | 836 | 23 | 2.8 | 37.6 | 2.0 | 3.6 | 238.0 | 10500 |
| 4,4-dibromobenzophenone | MITI | 2 | 647 | 2 | 0.3 | 6.5 | 0.2 | 2.2 | 10.8 | 1830000 |
| Acephate | S OP | 35 | 836 | 2 | 0.2 | 107.5 | 2.7 | 52.0 | 163.0 | 12000 |
| Acetamiprid | S NEO | 5 | 836 | 16 | 1.9 | 87.3 | 2.9 | 9.0 | 304.0 | 99000 |
| Aldicarb sulfone | S CARB | 10 | 836 | 27 | 3.2 | 38.0 | 0.7 | 17.0 | 97.2 | 2000000 |
| Aldicarb sulfoxide | S CARB | 20 | 836 | 43 | 5.1 | 398.0 | 11.4 | 13.4 | 1245.0 | 33600 |
| Allethrin | PYR | 1 | 836 | 16 | 1.9 | 19.4 | 1.1 | 1.7 | 139.0 | 48800 |
| Amicarbazone | HERB | 30 | 836 | 1 | 0.1 | 98.0 |  | 98.0 | 98.0 | 1120000 |
| Atrazine | S HERB | 1 | 836 | 107 | 12.8 | 13.1 | 0.6 | 1.0 | 154.0 | 980000 |
| Azinphos methyl | OP | 3 | 886 | 40 | 4.5 | 65.0 | 4.7 | 3.9 | 643.0 | 2420 |
| Azoxystrobin | S FUNG | 1 | 886 | 97 | 10.9 | 18.0 | 1.1 | 1.0 | 278.0 | 1120000 |
| Bendiocarb | S CARB | 2 | 885 | 2 | 0.2 | 13.8 | 0.4 | 5.5 | 22.0 | 2640 |
| Bifenthrin | PYR | 0.4 | 886 | 59 | 6.7 | 7.7 | 0.4 | 0.7 | 56.1 | 150 |
| Boscalid | S FUNG | 1 | 836 | 29 | 3.5 | 128.4 | 6.5 | 1.4 | 962.0 | 1550000 |
| Captan | FUNG | 10 | 886 | 54 | 6.1 | 372.3 | 45.4 | 16.0 | 10000.0 | 1080000 |
| Carbaryl | PS CARB | 5 | 836 | 44 | 5.3 | 170.2 | 9.9 | 4.5 | 1010.0 | 10500 |
| Carbendazim (benomyl) | S FUNG | 1 | 836 | 43 | 5.1 | 20.0 | 1.1 | 1.5 | 149.0 | 500000 |
| Carbofuran | S CARB | 5 | 836 | 1 | 0.1 | 5.8 |  | 5.8 | 5.8 | 1270 |
| Carbofuran, 3-hydroxy | S CARB | 3 | 836 | 4 | 0.5 | 10.4 | 0.3 | 3.6 | 21.1 | 1270 |
| Carfentrazone ethyl | PS HERB | 1 | 836 | 6 | 0.7 | 5.0 | 0.2 | 1.5 | 17.0 | 1140000 |
| Chlorfenapyr | PS MITI | 1 | 662 | 8 | 1.2 | 3.1 | 0.1 | 1.1 | 11.9 | 1200 |
| Chlorfenvinphos | OP | 6 | 647 | 2 | 0.3 | 21.7 | 0.6 | 10.7 | 32.7 | 23200 |
| Chlorferone (coumaphos) | OP | 25 | 647 | 12 | 1.9 | 1135.8 | 49.4 | 25.0 | 4390.0 | 46300 |
| Chlorothalonil | FUNG | 1 | 797 | 316 | 39.6 | 1860.4 | 286.3 | 1.0 | 98900.0 | 1110000 |
| Chlorpyrifos | OP | 0.1 | 886 | 396 | 44.7 | 40.1 | 4.0 | 0.1 | 890.0 | 1220 |
| Coumaphos | OP | 1 | 887 | 668 | 75.3 | 1454.6 | 174.8 | 1.0 | 91900.0 | 46300 |
| Coumaphos oxon | OP | 5 | 747 | 227 | 30.4 | 92.3 | 5.8 | 1.3 | 1300.0 | 46300 |
| Cyfluthrin | PYR | 1 | 886 | 57 | 6.4 | 8.1 | 0.2 | 1.1 | 44.7 | 220 |
| Cyhalothrin | PYR | 0.1 | 886 | 69 | 7.8 | 5.6 | 0.4 | 0.1 | 71.0 | 790 |
| Cypermethrin | PYR | 1 | 886 | 68 | 7.7 | 15.9 | 0.9 | 1.6 | 131.0 | 1350 |
| Cyphenothrin | PYR | 20 | 836 | 1 | 0.1 | 21.8 |  | 21.8 | 21.8 |  |
| Cyprodinil | S FUNG | 5 | 836 | 35 | 4.2 | 140.9 | 12.7 | 5.3 | 2150.0 | 3320000 |
| DDD-p,p' | OC | 4 | 836 | 2 | 0.2 | 12.6 | 0.0 | 11.8 | 13.4 | 52400 |
| DDE-p,p' | OC | 0.2 | 836 | 17 | 2.0 | 7.3 | 0.3 | 0.2 | 31.0 | 52400 |
| DDT-p,p' | OC | 2 | 836 | 2 | 0.2 | 20.8 | 0.7 | 6.0 | 35.6 | 52400 |
| Deltamethrin | PYR | 20 | 886 | 30 | 3.4 | 94.9 | 3.8 | 9.3 | 613.0 | 500 |
| Diazinon | OP | 0.1 | 835 | 39 | 4.7 | 7.8 | 0.3 | 0.1 | 29.0 | 2220 |
| Dichlorobenzene-para | OC | 6 | 170 | 9 | 5.3 | 137.9 | 26.2 | 6.9 | 1050.0 |  |
| Dicofol | OC | 0.4 | 886 | 65 | 7.3 | 13.2 | 0.8 | 0.4 | 143.0 | 370000 |
| Dieldrin | CYC | 4 | 886 | 7 | 0.8 | 40.5 | 2.3 | 6.9 | 195.0 | 2050 |
| Difenoconazole | S FUNG | 10 | 836 | 7 | 0.8 | 160.4 | 3.3 | 48.3 | 344.0 | 1260000 |
| Diflubenzuron | IGR | 10 | 836 | 4 | 0.5 | 75.0 | 1.8 | 15.0 | 128.0 | 460000 |
| Diphenamid | S FUNG | 1 | 836 | 2 | 0.2 | 1.0 | 0.0 | 1.0 | 1.0 | 24320000 |
| Dimethomorph | S FUNG | 15 | 647 | 12 | 1.9 | 54.6 | 1.9 | 16.0 | 166.0 | 308000 |
| Diphenylamine | FUNG | 2 | 189 | 6 | 3.2 | 13.2 | 0.8 | 3.6 | 32.0 |  |
| DMA (amitraz) | FORM | 50 | 648 | 62 | 9.6 | 799.0 | 37.2 | 120.0 | 4740.0 | 750000 |
| DMPF (amitraz) | FORM | 4 | 648 | 206 | 31.8 | 1327.7 | 174.8 | 6.0 | 43000.0 | 750000 |
| Endosulfan I | CYC | 0.1 | 886 | 218 | 24.6 | 9.7 | 0.5 | 0.4 | 95.0 | 78700 |
| Endosulfan II | CYC | 0.1 | 886 | 157 | 17.7 | 7.1 | 0.3 | 0.1 | 67.7 | 78700 |
| Endosulfan sulfate | CYC | 0.1 | 886 | 132 | 14.9 | 4.7 | 0.2 | 0.2 | 35.0 | 218000 |
| Esfenvalerate | PYR | 0.5 | 886 | 114 | 12.9 | 7.9 | 0.4 | 0.5 | 59.6 | 2240 |
| Ethion | OP | 2 | 836 | 2 | 0.2 | 107.3 | 1.2 | 83.6 | 131.0 | 107000 |
| Ethofumesate | S HERB | 5 | 836 | 2 | 0.2 | 392.0 | 8.2 | 224.0 | 560.0 | 500000 |
| Etoxazole | MITI | 1 | 836 | 1 | 0.1 | 1.2 |  | 1.2 | 1.2 | 2000000 |
| Famoxadone | FUNG | 20 | 836 | 6 | 0.7 | 98.3 | 0.9 | 73.5 | 141.0 | 625000 |
| Fenamidone | FUNG | 10 | 836 | 2 | 0.2 | 106.0 | 1.6 | 73.9 | 138.0 | 936000 |
| Fenbuconazole | S FUNG | 6 | 647 | 31 | 4.8 | 152.7 | 14.2 | 7.4 | 1970.0 | 1490000 |
| Fenhexamid | FUNG | 5 | 647 | 10 | 1.5 | 31.9 | 1.4 | 5.8 | 129.0 | 1580000 |
| Fenoxaprop-ethyl | S HERB | 6 | 836 | 1 | 0.1 | 15.4 |  | 15.4 | 15.4 | 1500000 |
| Fenpropathrin | PYR | 0.4 | 886 | 119 | 13.4 | 23.0 | 1.7 | 0.4 | 488.0 | 500 |
| Fipronil | INS | 1 | 836 | 6 | 0.7 | 522.8 | 43.0 | 1.1 | 3060.0 | 50 |
| Fluoxastrobin | S FUNG | 4 | 836 | 2 | 0.2 | 33.8 | 0.5 | 23.1 | 44.5 | 2000000 |
| Fluridone | S HERB | 5 | 836 | 5 | 0.6 | 9.7 | 0.3 | 5.7 | 24.0 | 3630000 |
| Flutolanil | S FUNG | 4 | 836 | 4 | 0.5 | 55.2 | 1.8 | 7.2 | 105.0 | 1330000 |
| Fluvalinate | PYR | 1 | 887 | 749 | 84.4 | 2947.6 | 330.7 | 1.1 | 204000.0 | 15860 |
| Heptachlor | CYC | 4 | 836 | 1 | 0.1 | 31.0 |  | 31.0 | 31.0 | 645000 |
| Heptachlor epoxide | CYC | 1 | 836 | 2 | 0.2 | 7.5 | 0.3 | 1.7 | 13.3 | 645000 |
| Hexachlorobenzene | FUNG | 0.1 | 886 | 8 | 0.9 | 0.3 | 0.0 | 0.1 | 1.0 | 4680 |
| Imidacloprid | S NEO | 2 | 836 | 14 | 1.7 | 77.5 | 5.9 | 2.4 | 646.0 | 280 |
| Imidacloprid olefin | S NEO | 25 | 836 | 2 | 0.2 | 597.0 | 2.1 | 554.0 | 640.0 | 280 |
| Imidacloprid, 5-hydroxy | S NEO | 25 | 836 | 2 | 0.2 | 151.0 | 0.0 | 150.0 | 152.0 | 280 |
| Indoxacarb | INS | 10 | 836 | 12 | 1.4 | 152.6 | 4.9 | 10.0 | 531.0 | 600000 |
| Iprodione | FUNG | 10 | 836 | 15 | 1.8 | 252.4 | 6.9 | 10.3 | 636.0 | 1020000 |
| Malathion | OP | 1 | 886 | 22 | 2.5 | 10.0 | 0.5 | 0.9 | 61.0 | 3950 |
| Metalaxyl | S FUNG | 1 | 832 | 1 | 0.1 | 1.4 |  | 1.4 | 1.4 | 1130000 |
| Methidathion | OP | 1 | 886 | 41 | 4.6 | 17.6 | 0.4 | 1.0 | 78.7 | 2010 |
| Methoxyfenozide | IGR | 0.4 | 836 | 77 | 9.2 | 59.3 | 2.9 | 0.4 | 495.0 | 1000000 |
| Metolachlor | PS HERB | 2 | 836 | 57 | 6.8 | 15.7 | 0.9 | 2.6 | 149.0 | 1260000 |
| Metribuzin | S HERB | 1 | 836 | 20 | 2.4 | 6.1 | 0.3 | 1.0 | 44.0 | 567000 |
| Myclobutanil | S FUNG | 2 | 836 | 20 | 2.4 | 159.9 | 8.6 | 4.4 | 981.0 | 1870000 |
| Norflurazon | S HERB | 1 | 836 | 32 | 3.8 | 19.3 | 0.8 | 1.1 | 108.0 | 1630000 |
| Oxamyl | S CARB | 5 | 836 | 4 | 0.5 | 32.0 | 0.3 | 20.0 | 43.0 | 31100 |
| Oxyfluorfen | HERB | 0.5 | 886 | 25 | 2.8 | 8.0 | 0.3 | 0.5 | 34.0 | 1000000 |
| Parathion methyl | OP | 1 | 836 | 6 | 0.7 | 3.2 | 0.1 | 1.5 | 6.1 | 1820 |
| Pendimethalin | HERB | 1 | 662 | 185 | 27.9 | 43.7 | 5.8 | 1.1 | 1730.0 | 665000 |
| Permethrin | PYR | 10 | 886 | 27 | 3.0 | 1594.3 | 184.8 | 9.6 | 21700.0 | 1120 |
| Phenothrin | PYR | 10 | 836 | 1 | 0.1 | 83.9 |  | 83.9 | 83.9 |  |
| Phosalone | OP | 10 | 647 | 1 | 0.2 | 31.3 |  | 31.3 | 31.3 | 74600 |
| Phosmet | OP | 2 | 886 | 28 | 3.2 | 213.5 | 21.3 | 2.9 | 3390.0 | 8030 |
| Piperonyl butoxide | SYN | 6 | 836 | 2 | 0.2 | 119.6 | 4.3 | 31.1 | 208.0 | 180000 |
| Pirimiphos methyl | OP | 4 | 836 | 1 | 0.1 | 57.0 |  | 57.0 | 57.0 | 3230 |
| Potasan (coumaphos) | OP | 10 | 647 | 8 | 1.2 | 91.1 | 2.3 | 14.5 | 160.0 | 46300 |
| Prallethrin | PYR | 4 | 836 | 7 | 0.8 | 6.5 | 0.1 | 4.3 | 8.6 | 280 |
| Pronamide | S HERB | 1 | 836 | 22 | 2.6 | 64.3 | 3.7 | 1.7 | 378.0 | 1580000 |
| Propanil | HERB | 10 | 836 | 2 | 0.2 | 311.5 | 2.3 | 265.0 | 358.0 | 1320000 |
| Propiconazole | S FUNG | 3 | 836 | 16 | 1.9 | 121.1 | 3.5 | 3.1 | 361.0 | 625000 |
| Pyraclostrobin | FUNG | 1 | 836 | 15 | 1.8 | 79.6 | 4.2 | 1.8 | 438.0 | 870000 |
| Pyrethrins | PYR | 20 | 836 | 20 | 2.4 | 72.6 | 1.8 | 19.0 | 222.0 | 1480 |
| Pyridaben | MITI | 1 | 836 | 6 | 0.7 | 16.6 | 0.3 | 5.4 | 26.6 | 240 |
| Pyrimethanil | FUNG | 2 | 836 | 26 | 3.1 | 16.2 | 0.8 | 2.0 | 83.0 | 1000000 |
| Pyriproxyfen | IGR | 1 | 836 | 2 | 0.2 | 4.9 | 0.1 | 2.2 | 7.6 | 1000000 |
| Quintozene = PCNB | FUNG | 1 | 836 | 3 | 0.4 | 1.6 | 0.0 | 1.0 | 2.5 | 500000 |
| Sethoxydim | S HERB | 1 | 836 | 2 | 0.2 | 90.6 | 4.0 | 8.2 | 173.0 | 100000 |
| Simazine | S HERB | 5 | 836 | 13 | 1.6 | 21.1 | 0.5 | 5.2 | 54.0 | 967000 |
| Spirodiclofen | MITI | 1 | 836 | 2 | 0.2 | 15.2 | 0.7 | 1.9 | 28.5 | 1960000 |
| Spiromesifen | S INS | 10 | 836 | 1 | 0.1 | 10.0 |  | 10.0 | 10.0 | 3140000 |
| Tebuconazole | S FUNG | 3 | 836 | 5 | 0.6 | 31.5 | 0.8 | 6.4 | 65.0 | 1100000 |
| Tebufenozide | IGR | 2 | 836 | 39 | 4.7 | 17.1 | 0.9 | 2.0 | 164.0 | 2340000 |
| Tebuthiuron | S HERB | 1 | 836 | 11 | 1.3 | 16.0 | 0.5 | 1.6 | 48.0 | 650000 |
| Tefluthrin | PYR | 1 | 836 | 1 | 0.1 | 3.3 |  | 3.3 | 3.3 | 2800 |
| Tetradifon | MITI | 1 | 836 | 2 | 0.2 | 7.9 | 0.2 | 4.7 | 11.1 | 110000 |
| Tetramethrin | PYR | 6 | 836 | 4 | 0.5 | 17.5 | 0.3 | 6.1 | 23.0 | 1550 |
| Thiabendazole (thiophanate-Me) | S FUNG | 1 | 836 | 12 | 1.4 | 10.8 | 0.7 | 1.4 | 76.0 | 500000 |
| Thiacloprid | S NEO | 1 | 836 | 26 | 3.1 | 22.2 | 1.0 | 1.7 | 115.0 | 252000 |
| Thiamethoxam | S NEO | 5 | 836 | 1 | 0.1 | 53.3 |  | 53.3 | 53.3 | 150 |
| THPI (captan) | PS FUNG | 30 | 647 | 39 | 6.0 | 227.5 | 9.3 | 37.7 | 1550.0 | 1080000 |
| Triadimefon | S FUNG | 2 | 836 | 1 | 0.1 | 2.4 |  | 2.4 | 2.4 | 250000 |
| Tribufos = DEF | SYN | 2 | 836 | 11 | 1.3 | 18.1 | 0.5 | 1.4 | 59.0 | 442000 |
| Trifloxystrobin | PS FUNG | 0.5 | 886 | 57 | 6.4 | 132.9 | 14.3 | 0.6 | 2610.0 | 1750000 |
| Trifluralin | HERB | 1 | 647 | 34 | 5.3 | 3.7 | 0.3 | 1.0 | 36.0 | 685000 |
| Vinclozolin | FUNG | 1 | 886 | 19 | 2.1 | 6.5 | 0.3 | 1.0 | 27.0 | 1000000 |

| **^#^**Class: CAR = carbamate, CYC = cyclodiene, FORM = formamidine, FUNG = fungicide, HERB = herbicide, IGR = insect growth regulator, INS = misc. insecticide, MITI = miticide, NEO = neonicotinoid, OC = organochlorine, OP = organophosphate, PS = partial systemic, PYR = pyrethroid, S = systemic |
| --- |
| **^†^**LOD = limit of detection (ppb).  **^§^**Mean and SEM for detections > LOD. |
| **^¶^**LD50 is ave. honey bee acute toxicity from literature in ppb = 10,000 X µg/bee; bold nos. < LD50 |
