# Supplementary material for: High Levels of Miticides and Agrochemicals in North American Apiaries: Implications for Honey Bee Health
Source: PLoS One. 2010 Mar 19;5(3):e9754. doi: 10.1371/journal.pone.0009754 (PMC2841636; doi:10.1371/journal.pone.0009754)
Supplement: Table S2 — Summary of pesticides and their metabolites not detected in 887 North American beehive and related samples. (0.20 MB DOC) [file pone.0009754.s002.doc]

**Table S2.** Summary of pesticides and their metabolites not

detected in 887 North American beehive and related samples.

| **Pesticide or Metabolite** | **Class^#^** | **LOD^†^** | **Samples Analyzed** |
| --- | --- | --- | --- |
| Acetochlor | S HERB | 10 | 647 |
| Alachlor | S HERB | 10 | 836 |
| Aldicarb | S CARB | 4 | 836 |
| Aldrin | CYC | 10 | 836 |
| Amitraz | FORM | 4 | 836 |
| Benoxacor | HERB | 4 | 836 |
| BHC-alpha | OC | 4 | 836 |
| Bifenazate | MITI | 20 | 836 |
| Bromuconazole | S FUNG | 20 | 836 |
| Buprofezin | S FUNG | 20 | 836 |
| Carboxin | S FUNG | 4 | 836 |
| Chloroneb | S FUNG | 4 | 89 |
| Chlorothalonil, hydroxy | S FUNG | 50 | 544 |
| Chlorpropham | HERB | 6 | 836 |
| Chlorpyrifos methyl | OP | 1 | 836 |
| Chlorpyrifos methyl oxon | OP | 10 | 89 |
| Chlorpyrifos oxon | OP | 10 | 100 |
| Clofentezine | MITI | 10 | 836 |
| Clothianidin | S NEO | 5 | 836 |
| Cyproconazole | S HERB | 40 | 89 |
| Cyromazine | INS | 85 | 89 |
| Dichlorvos | OP | 10 | 836 |
| Dicloran | FUNG | 1 | 478 |
| Dimethenamid | PS HERB | 10 | 647 |
| Dimethoate | S OP | 20 | 836 |
| Dinotefuran | S NEO | 10 | 836 |
| Endrin | CYC | 10 | 647 |
| Epoxiconazole | FUNG | 1 | 836 |
| Etridiazole | FUNG | 6 | 836 |
| Fenamiphos | S OP | 20 | 89 |
| Fenpyroximate | MITI | 5 | 648 |
| Fenthion | OP | 10 | 692 |
| Fenthion oxon | OP | 20 | 89 |
| Fenthion sulfone | OP | 30 | 89 |
| Flonicamid | S INS | 40 | 144 |
| Fluazifop | S HERB | 45 | 89 |
| Flucarbazone | S HERB | 50 | 89 |
| Fludioxonil | PS FUNG | 10 | 836 |
| Fluroxypyr methyl heptyl | HERB | 2 | 89 |
| Hexaconazole | S FUNG | 40 | 89 |
| Hexythiazox | MITI | 6 | 836 |
| Hydroprene | IGR | 10 | 836 |
| Imazalil | S FUNG | 5 | 836 |
| Lindane | OC | 4 | 836 |
| Linuron | S HERB | 50 | 836 |
| Malathion oxon | OP | 20 | 189 |
| Methamidophos | S OP | 9 | 836 |
| Methidathion oxon | OP | 5 | 89 |
| Methomyl | S CARB | 10 | 836 |
| MGK-264 | SYN | 10 | 836 |
| MGK-326 | SYN | 10 | 836 |
| Omethoate | S OP | 12 | 89 |
| Pentachloroaniline | OC | 1 | 89 |
| Pentachlorobenzene | OC | 0.1 | 89 |
| Pentachlorothioanisole | OC | 0.2 | 89 |
| Perthane | OC | 20 | 89 |
| Phorate | S OP | 4 | 836 |
| Phorate oxon | S OP | 20 | 89 |
| Profenofos | OP | 10 | 836 |
| Propachlor | S HERB | 10 | 836 |
| Propargite | MITI | 10 | 836 |
| Propazine | S HERB | 4 | 647 |
| Propetamphos | OP | 4 | 647 |
| Propham | S HERB | 20 | 692 |
| Propoxycarbazone | S HERB | 30 | 89 |
| Pymetrozine | S INS | 20 | 144 |
| Pyrazon = Pyramin | S HERB | 6 | 89 |
| Quinoxyfen | S FUNG | 10 | 647 |
| Quizalofop | S HERB | 20 | 89 |
| Resmethrin | PYR | 10 | 647 |
| Tepraloxydim | S HERB | 5 | 89 |
| Terbacil | HERB | 20 | 89 |
| Tetrachlorvinphos | OP | 4 | 836 |
| Tetraconazole | S FUNG | 6 | 836 |
| Thiabendazole, 5-hydroxy | S FUNG | 25 | 89 |
| Thidazuron | HERB | 25 | 89 |
| Thiobencarb | HERB | 40 | 89 |
| Triadimenol | PS FUNG | 45 | 836 |
| Triasulfuron | S HERB | 5 | 89 |
| Triflumizole | S FUNG | 10 | 836 |
| Triticonazole | FUNG | 10 | 832 |

**^#^**Class: CAR = carbamate, CYC = cyclodiene, FORM = formamidine, FUNG = fungicide, HERB = herbicide, IGR = insect growth regulator, INS = misc. insecticide, MITI = miticide, NEO = neonicotinoid, OC = organochlorine, OP = organophosphate, PS = partial systemic, PYR = pyrethroid, S = systemic

**^†^**LOD = limit of detection (ppb).
